# The impact of trauma relevant concentrations of prostaglandin E_2_ on the anti-microbial activity of the innate immune system

**DOI:** 10.3389/fimmu.2024.1401185

**Published:** 2024-10-22

**Authors:** Thomas Nicholson, Antonio Belli, Janet M. Lord, Jon Hazeldine

**Affiliations:** ^1^ Institute of Inflammation and Ageing, University of Birmingham, Birmingham, United Kingdom; ^2^ National Institute for Health Research Surgical Reconstruction and Microbiology Research Centre, Queen Elizabeth Hospital Birmingham, Birmingham, United Kingdom; ^3^ Medical Research Council (MRC)-Versus Arthritis Centre for Musculoskeletal Ageing Research, University of Birmingham, Birmingham, United Kingdom

**Keywords:** critical care, immunosuppression, innate immunity, prostaglandin E_2_, trauma

## Abstract

**Background:**

The mechanisms underlying the state of systemic immune suppression that develops following major trauma are poorly understood. A post-injury increase in circulating levels of prostaglandin E_2_ (PGE_2_) has been proposed as a contributory factor, yet few studies have addressed how trauma influences PGE_2_ biology.

**Methods:**

Blood samples from 95 traumatically-injured patients (injury severity score ≥8) were collected across the pre-hospital (≤2 hours), acute (4-12 hours) and subacute (48-72 hours) post-injury settings. Alongside *ex vivo* assessments of lipopolysaccharide (LPS)-induced cytokine production by monocytes, neutrophil reactive oxygen species production and phagocytosis, serum concentrations of PGE_2_ and its scavenger albumin were measured, and the expression of enzymes and receptors involved in PGE_2_ synthesis and signalling analysed. Leukocytes from trauma patients were treated with cyclooxygenase (COX) inhibitors (indomethacin or NS-398), or the protein kinase A inhibitor H89, to determine whether injury-induced immune suppression could be reversed by targeting the PGE_2_ pathway. The effect that trauma relevant concentrations of PGE_2_ had on the anti-microbial functions of neutrophils, monocytes and monocyte-derived macrophages (MDMs) from healthy controls (HC) was examined, as was the effect of PGE_2_ on efferocytosis. To identify factors that may trigger PGE_2_ production post-trauma, leukocytes from HC were treated with mitochondrial-derived damage associated molecular patterns (mtDAMPs) and COX-2 expression and PGE_2_ generation measured.

**Results:**

PGE_2_ concentrations peaked in blood samples acquired ≤2 hours post-injury and coincided with significantly reduced levels of albumin and impaired LPS-induced cytokine production by monocytes. Significantly higher COX-2 and phospholipase A_2_ expression was detected in neutrophils and/or peripheral blood mononuclear cells isolated from trauma patients. Treatment of patient leukocytes with indomethacin, NS-398 or H89 enhanced LPS-induced cytokine production and neutrophil extracellular trap generation. Exposure to physiological concentrations of PGE_2_ suppressed the anti-microbial activity of monocytes, neutrophils and MDMs of HC, but did not influence efferocytosis. In a formyl-peptide receptor-1 dependent manner, mtDAMP treatment significantly increased COX-2 protein expression in neutrophils and monocytes, which resulted in increased PGE_2_ production.

**Conclusions:**

Physiological concentrations of PGE_2_ suppress the anti-microbial activities of neutrophils, monocytes and MDMs. Targeting the PGE_2_ pathway could be a therapeutic approach by which to enhance innate immune function post-injury.

## Introduction

Traumatic injury triggers two opposing clinical syndromes: a systemic inflammatory response syndrome (SIRS) and a compensatory anti-inflammatory response syndrome (CARS). Aimed at restoring immunological homeostasis, features of the CARS response are detected within minutes of injury and include decreased production of pro-inflammatory cytokines by lipopolysaccharide (LPS) challenged monocytes and reduced anti-microbial activity of neutrophils (1–4). Resulting in a state of systemic immune suppression, severe trauma triggers prolonged and/or exaggerated CARS responses that increase the susceptibility of the injured patient to the development of hospital acquired infections (HAIs) (5). With incidence rates as high as 49.8%, pneumonia and urinary tract infections (UTIs) are common secondary complications following major trauma, and represent a significant cause of patient morbidity and mortality (6–9). Indeed, subjects who develop pneumonia or UTIs experience significantly longer intensive care unit lengths of stay, are more likely to be discharged to rehabilitation facilities, exhibit poor long-term physiological recovery and are at an increased risk of in-hospital mortality when compared to their non-infected counterparts (9–12). Predisposing patients to the development of HAIs, a dysregulated CARS response is of clinical significance (5), yet the mechanisms that underpin trauma-induced suppression of innate immune responses are poorly understood.

Generated from the membrane phospholipid arachidonic acid (AA) via the actions of the enzymes phospholipase A2 (cPLA_2_), cyclooxygenase-2 (COX-2) and microsomal PGE synthase-1 (mPGES-1), prostaglandin E_2_ (PGE_2_) is a potent immunomodulatory molecule (13). By binding to the G_αs_ protein coupled E prostanoid (EP) receptors EP2 and EP4, PGE_2_ activates adenylate cyclase, resulting in elevated intracellular levels of the immunosuppressive second messenger cyclic adenosine monophosphate (cAMP) (13). Through this pathway, PGE_2_ has been shown to inhibit LPS-induced pro-inflammatory cytokine production by monocytes (14, 15) and to suppress a range of neutrophil anti-microbial defence mechanisms, which include phagocytosis, reactive oxygen species (ROS) generation and the formation of extracellular traps (16–19). In times of severe inflammation, it has been hypothesised that increased synthesis, reduced catabolism and/or increased bioavailability of PGE_2_ may be pathogenic (20). Termed “injurious resolution”, this theory proposes that due to its immune suppressive properties, elevated levels of PGE_2_ would be deleterious for host immunity and thus increase the susceptibility of critically ill patients to HAIs (20).

In the settings of traumatic brain injury (TBI) and burns, prospective studies have measured elevated plasma concentrations of PGE_2_ for up to three weeks post-injury (21–23). Suggesting that leukocytes are a source of this PGE_2_, significantly higher mRNA and/or protein expression of COX-2 has been detected in neutrophils and peripheral blood mononuclear cells (PBMCs) isolated from trauma and burns patients, with these cells generating significantly more PGE_2_ *ex vivo* when compared to leukocytes from uninjured controls (24–29). Implicating PGE_2_ in promoting trauma-induced immune suppression, pre-treatment of PBMCs isolated from injured patients with the COX inhibitor indomethacin enhances the functional responses of T cells to *ex vivo* stimulation (27, 30), whilst murine studies have demonstrated that inhibition of COX-2 following burn or traumatic injury can improve survival rates upon infectious challenge (31, 32). Taken together, these data highlight how major trauma modulates PGE_2_ biology. However, there remain many unanswered questions. For example, *(1) What are the factors generated post-injury that promote increased expression of COX-2 in leukocytes* and *(2) Can ex vivo inhibition of COX signalling enhance the functional responses of innate immune cells post-trauma?* Furthermore, it should be noted that *in vitro* studies that have reported upon the immune suppressive effects of PGE_2_ have used concentrations ranging between 3,528-3,528,000 pg/ml (16–19, 33), which exceed those measured in the circulation of trauma patients (600-2,000 pg/ml) (21, 22). This therefore raises the question as to whether PGE_2_ at physiological doses can induce the functional impairments in innate immunity that occur during the post-injury CARS response.

Recently, it was suggested that the CARS response induced by major injury should be regarded as a disorder caused by the hyper-resolution of systemic inflammation (34). Mechanistically, it was proposed that the generation of suppressive molecules, so-called “suppressive/inhibiting inducible damage-associated molecular patterns” (SAMPs), leads to an exaggerated and long-lasting CARS response that renders the trauma patient susceptible to HAIs (34). At the cellular level, it was implied that SAMPs are produced in the immediate post-injury phase by damage-associated molecular pattern (DAMP) activated innate immune cells (34). Of note, PGE_2_ was mentioned as a potential SAMP (34). Interestingly, *in vitro* studies have shown that exposure to the bacterial-derived peptide N-Formylmethionine-leucyl-phenylalanine (fMLP), which activates the same signalling pathways as mitochondrial-derived N-formylated peptides, can increase COX-2 expression and PGE_2_ synthesis by neutrophils (35–37). Given that mitochondrial-derived DAMPs (mtDAMPs) are present in the circulation of trauma patients within minutes of injury (2, 4), and are potent activators of neutrophils (38–40), it is conceivable that these molecules could promote immune suppression by up-regulating COX-2 expression and PGE_2_ synthesis by circulating leukocytes. However, to our knowledge, no study to date has explored this possibility.

With the aim of investigating how PGE_2_ influences innate immunity post-trauma, we have measured circulating PGE_2_ levels and analysed the expression of enzymes and receptors involved in PGE_2_ synthesis and signalling in neutrophils and PBMCs isolated from adult trauma patients during the ultra-early (≤120 minutes) and acute (4–72 hours) post-injury phases. Moreover, to determine whether manipulation of the PGE_2_ pathway could enhance innate immune function post-trauma, we have investigated the effect that inhibition of cyclooxygenases has on *ex vivo* neutrophil extracellular trap (NET) formation and the production of pro-inflammatory cytokines by LPS-challenged monocytes. Alongside these studies, we have examined how PGE_2_, at physiological concentrations, influences the anti-microbial activities of neutrophils, monocytes and monocyte derived macrophages (MDMs) from healthy volunteers, and investigated the effect that exposure to mtDAMPs has on COX-2 expression and/or PGE_2_ production by neutrophils and monocytes.

## Materials and methods

### Study setting

This manuscript presents data acquired from subjects enrolled into the Brain Biomarkers after Trauma Study, an ongoing prospective observational study of adult trauma patients conducted at a single Major Trauma Centre in the UK (University Hospitals Birmingham NHS Foundation Trust, Birmingham). Ethical approval for the study was granted by the North Wales Research Ethics Committee West (REC reference: 13/WA/0399, protocol number: RG_13–164).

Patient enrolment began in the pre-hospital setting, where on a 24/7 basis between March 2016 and May 2023, emergency care teams acquired blood samples from adult trauma patients (≥18 years) with a suspected injury severity score (ISS) ≥8. Details on pre-hospital enrolment, study exclusion criteria and patient consent have been described previously (3).

### Blood sampling

Peripheral venous blood samples were acquired from trauma patients at three post-injury time-points: pre-hospital, 4-12 and 48-72 hours. Blood samples were collected into BD Vacutainers^®^ (BD Biosciences, Oxford, UK) containing either ethylenediaminetetraacetic acid (EDTA), lithium heparin, z-serum clotting activator or a 1/10 volume of 3.2% trisodium citrate. For samples acquired in the pre-hospital setting, vacutainers were stored at room temperature (RT) during their transportation to hospital and collected for analysis within 1 hour of arrival at the accident and emergency department by a single laboratory researcher. Processing of in-hospital blood samples began within 1 hour of acquisition by the same laboratory researcher.

Sixty-seven adults (mean age 30 years, range 19–60 years) served as a cohort of healthy controls (HC). In accordance with the Declaration of Helsinki and the ethical approval granted by the University of Birmingham Research Ethics Committee (Refs: ERN_12-1184), all HC provided written informed consent prior to blood sampling. HC were defined as subjects who were not taking any regular medication for a diagnosed illness and did not have an acute episode of infection prior to study inclusion.

Full blood counts were performed using a Sysmex XN-1000 differential haematology analyser (Sysmex UK, Milton Keynes, UK) that measures a white cell differential. The analyser uses fluorescence dyes that label intracellular DNA and RNA, with the intensity of the fluorescence signal directly proportional to the nucleic acid content of the cell. Daily internal quality control measurements (XN check, Sysmex UK) and monthly external quality control samples (UKNEQAS, Watford, UK) ensured instrument performance.

Serum was prepared from blood collected into BD vacutainers containing z-serum clotting activator. Following a 30 minute incubation at RT, blood samples were centrifuged at 1,620 x g for 10 minutes at 4°C, after which serum was aliquoted and stored at −80°C until analysed.

### Isolation of neutrophils, monocytes and PBMCs

Neutrophils were isolated by Percoll density gradient centrifugation (Merck Life Science UK Limited, Dorset, UK). Determined by the use of a Sysmex XN-1000 automated differential haematology analyser, purity of neutrophil preparations were routinely ≥98% (**Supplementary Figure 1**). Post-isolation, neutrophils were re-suspended at concentrations of 1-5x10^6^/ml in RPMI-1640 media supplemented with 2 mM L-glutamine, 100 U/ml penicillin and 100 µg/ml streptomycin (GPS; Merck Life Science UK Limited) or RPMI-1640 media supplemented with GPS and 10% heat-inactivated fetal calf serum [hereafter referred to as complete media (CM)].

PBMCs were isolated from citrate anti-coagulated blood by Percoll density gradient centrifugation or from heparin anti-coagulated blood by centrifugation on Ficoll-Paque™ PLUS gradients (GE Healthcare, Uppsala, Sweden). Post-isolation, PBMCs (5x10^6^) from citrate anti-coagulated blood were re-suspended in 1 ml of TRIzol reagent (Life Technologies, Cheshire, UK) and stored at -80°C prior to RNA extraction. PBMCs isolated from heparin anti-coagulated blood were re-suspended in CM for stimulation assays or in magnetic assisted cell sorting (MACS^®^) buffer (phosphate buffered saline (PBS) containing 0.5% bovine serum albumin (BSA) and 2 mM EDTA) for the isolation of monocytes by negative selection using MACS^®^ technology (pan monocyte isolation kit, Miltenyi BioTec, Gladbach, Germany). Post-isolation, the purity of monocyte preparations were determined using the Sysmex XN-1000 haematology analyser, and were routinely ≥96% (**Supplementary Figure 1**).

### Generation of MDMs

MDMs were generated from monocytes isolated from PBMCs using a Lymphoprep™ gradient (STEMCELL Technologies UK Ltd, Cambridge, UK) and adherence technique. PBMCs were prepared from heparinised whole blood and resuspended in serum free RPMI 1640 media supplemented with GPS. 4×10^6^ PBMCs were seeded into 6-well plates to prepare MDMs from which supernatants were collected, whilst 5×10^5^ PBMCs were seeded into 24-well plates for efferocytosis assays and phenotyping. Plated cells were allowed to adhere for 2 hours (37°C, 5% CO_2_) prior to culture in feeding media (RPMI 1640 media supplemented with 2 ng/ml granulocyte macrophage colony stimulating factor (R and D Systems, Abingdon, UK), 10% FCS and GPS) for a total of 10 days, with feeding media replaced every 3 days. Once differentiated into MDMs, (confirmed by morphology), cells were cultured in RPMI media with 10% FCS and GPS for one day prior to treatments.

### Measurements of PGE_2_, albumin, high mobility group box-1, lactate dehydrogenase, tumour necrosis factor-alpha and IL-10 concentrations

PGE_2_ concentrations in serum and cell free culture supernatants were quantified using a PGE_2_ parameter assay kit in accordance with manufacturer’s instructions (R and D Systems). Serum concentrations of HMGB-1 and LDH were measured using a commercially available enzyme linked immunosorbent assay (ELISA; IBL International, Hamburg, Germany) or activity assay kit (Merck Life Science UK Limited) respectively in line with manufacturer’s guidelines. Albumin concentrations in serum samples were measured on a Roche/Hitachi Cobas C system following manufacturer’s guidelines outlined in the Albumin Gen 2 kit (Roche, Basel, Switzerland). Concentrations of TNF-α and IL-10 in cell free culture supernatants were determined using commercially available DuoSet ELISA kits in line with manufacturer’s instructions (R and D Systems).

### Preparation of mitochondrial-derived damage associated molecular patterns

MtDAMPs were prepared from mitochondria isolated from K562 cells (ATCC^®^, Teddington, Middlesex, UK) as described previously (38). The protein content of mtDAMP preparations was determined by spectrophotometry (Nanodrop 2000; ThermoFisher Scientific, Paisley, UK).

### PGE_2_ treatments

To investigate the effect of exogenous PGE_2_ on immune function, heparinised whole blood samples or purified neutrophils were pre-treated for 30 minutes (37°C/5%CO_2_) with four doses of PGE_2_ (Merck Life Science UK Limited) or vehicle control. The doses of PGE_2_ were: (1) 600 pg/ml, the average concentration of PGE_2_ measured in 35 plasma samples obtained from patients with severe TBI on day 1 of injury (21); (2) 1,500 pg/ml, the mean PGE_2_ concentration measured in serum samples obtained from 52 traumatically-injured patients within 1-hour of injury; (3) 2,000 pg/ml, the concentration of PGE_2_ detected in 24 plasma samples of severe burns patients (22) and (4) 10,000 pg/ml, the concentration of PGE_2_ measured in inflammatory exudates obtained from a human model of self-resolving acute inflammation (41). To prevent endogenous production of PGE_2_, whole blood samples or neutrophils were treated for 1 hour (37°C/5%CO_2_) with 10 µM of the COX inhibitor indomethacin (Merck Life Science UK Limited) prior to stimulation.

To study the effect of PGE_2_ treatment on monocyte transcription, 5x10^5^ freshly isolated purified human monocytes, re-suspended in hanks balanced salt solution containing calcium and magnesium (HBSS^+/+^; Gibco, ThermoFisher Scientific), were treated for 1 hour with 10 µM indomethacin, followed by a 30 minute culture with 1,500 pg/ml PGE_2_ or vehicle control and a 2 hour stimulation with 100 ng/ml LPS. Post-treatment, cells were pelleted (1,500 x g, 2 minutes) and re-suspended in 350 µl RLT lysis buffer in preparation for RNA isolation using the commercially available RNeasy isolation kit (Qiagen, Manchester, UK).

For MDM experiments that measured cytokine concentrations in cell free supernatants and surface CD215 expression, MDMs were treated for 1 hour with 10 µM indomethacin or vehicle control prior to a 1 hour treatment with 10,000 pg/ml PGE_2_ or vehicle. MDMs were then stimulated with 100 ng/ml LPS for 4 hours, after which cell free supernatants were collected for ELISAs, and cells prepared for flow cytometric analysis. For efferocytosis assays and the determination of signal regulatory protein-alpha (SIRP-α) expression, MDMs were treated with 10,000 pg/ml PGE_2_ or vehicle for 1 hour prior to a 2 hour co-culture with neutrophils for efferocytosis assays or RPMI-1640 media for phenotypic profiling. Experimental protocols for each of these assays are detailed below.

### LPS stimulation of whole blood and quantification of TNF-α concentration

To determine how traumatic injury impacted upon TNF-α production by whole blood leukocytes, 400 µl aliquots of heparinised blood were stimulated with 100 ng/ml LPS (*E.Coli*, serotype 0111:B4; Merck Life Science UK Limited) or vehicle control for 4 or 18 hours (37°C/5%CO_2_). To examine whether *ex vivo* inhibition of cyclooxygenases altered leukocyte TNF-α production post-trauma, 400 µl aliquots of heparinised blood were treated for 1 hour (37°C/5%CO_2_) with 10 µM indomethacin or vehicle control prior to LPS stimulation (4 hours; 100 ng/ml; 37°C/5%CO_2_). To investigate the effect of exogenous PGE_2_ on TNF-α production, 400 µl aliquots of heparinised whole blood from HC were treated for 1 hour with 10 µM indomethacin in the presence or absence of 10 µM AH6809, 10 µM L161,982 or 10 µM rolipram (Merck Life Science UK Limited), after which, samples were incubated for 30 minutes with PGE_2_ (600-10,000 pg/ml) or vehicle control. Post-treatment, samples were stimulated for 4 hours with 100 ng/ml LPS (37°C/5%CO_2_). To determine the effect of adenylate cyclase activation on TNF-α production, heparinised blood samples were treated for 1 hour with 10 µM indomethacin, 100 µM forskolin (Merck Life Science UK Limited) and/or 10 µM rolipram prior to LPS stimulation (4 hours; 100 ng/ml; 37°C/5%CO_2_).

Following the above treatments, samples were centrifuged at 461 x g for 8 minutes at 4°C, after which cell free supernatants were collected and stored at -80°C until analysed. TNF-α concentrations were quantified using a human TNF-α DuoSet ELISA according to manufacturer’s instructions (R&D Systems) and normalised to monocyte counts.

### Neutrophil oxidative burst and phagocytic activity

Following manufacturer’s instructions of the commercially available PhagoBURST™ kit (BD Biosciences, Oxford, UK), neutrophil ROS production following stimulation with opsonised *E.coli* (multiplicity of infection, 1:40) or 1.62 µM phorbol 12-myristate 13-acetate (PMA) was measured in 100 µl aliquots of heparinised whole blood. For studies that investigated neutrophil function post-trauma, patient white blood cell counts were normalised to those of HC prior to testing in order to account for post-injury leukocytosis. To examine the effect of PGE_2_ treatment on ROS production, blood samples were treated with 10 µM indomethacin for 1 hour (37°C/5%CO_2_) followed by a 30 minute incubation with 1,500 pg/ml PGE_2_ or vehicle control (37°C/5%CO_2_) prior to PMA stimulation. For data analysis, 10,000 neutrophils, gated according to their forward scatter (FS)/sideward (SS) properties, were acquired on a BD FACSVia™ flow cytometer and data examined using CFlow software (BD Biosciences). For ROS production, data are presented as mean fluorescence intensity (MFI), with the values from untreated controls subtracted from *E.coli* and PMA stimulated samples. The flow cytometry gating strategy used for data acquisition and analysis is displayed in **Supplementary Figures 2A–D**.

To study the effect of traumatic injury on neutrophil phagocytosis, the commercially available PhagoTEST™ kit (BD Biosciences) was used. Following manufacturer’s guidelines, the ability of neutrophils to phagocytose opsonised *E.coli* (multiplicity of infection, 1:80) was measured in 100 µl aliquots of heparinised whole blood. Patient white blood cell counts were normalised to those of HC prior to testing in order to account for post-injury leukocytosis. For data analysis, 10,000 neutrophils, gated according to their FS/SS properties, were acquired on a BD FACSVia™ flow cytometer and data examined using CFlow software (BD Biosciences). Data are presented as phagocytic index, which was calculated as: (% phagocytosing neutrophils/100) X MFI. The flow cytometry gating strategy used for data acquisition and analysis is displayed in **Supplementary Figures 2E–H**.

To investigate the effect of PGE_2_ treatment on neutrophil phagocytic activity, the commercially available IngoFlowEx Kit was used (ExBio, Praha, Czech Republic). 50 µl heparinised blood samples were treated with 10 µM indomethacin for 1 hour (37°C/5%CO_2_) followed by a 60 minute incubation with 1,500 pg/ml PGE_2_ or vehicle control (37°C/5%CO_2_) prior to a 20 minute incubation (37°C) with opsonised *E.coli* (multiplicity of infection, 1:20). Phagocytosis of bacteria was assessed as described in manufacturer’s instructions. For analysis, 10,000 neutrophils, gated according to their FS/SS properties, were acquired on a MACSQuant Analyzer 10 flow cytometer (Miltenyi BioTec) and data examined using FlowJo software (BD Biosciences). Data are presented as phagocytic index, which was calculated as: (% phagocytosing neutrophils/100) X MFI.

### NET formation

Prior to the assessment of NET formation, neutrophils (2x10^5^ in RPMI + GPS) isolated from EDTA anti-coagulated blood samples were subjected to one of two treatment protocols: (1) 1 hour treatment with 10 µM indomethacin followed by a 30 minute incubation with 1,500 pg/ml PGE_2_ or vehicle control or (2) 1 hour treatment with the COX-2 specific inhibitor NS398 (0.1-1 µM; Merck Life Science UK Limited) or the protein kinase inhibitor H89 (500 nM, 10 or 20 µM; Merck Life Science UK Limited).

Following the treatments outlined above, neutrophils were stimulated with 25 nM PMA (Merck Life Science UK Limited) or vehicle control for 3 hours at 37°C/5%CO_2_. Post-incubation, supernatants were collected and centrifuged at 2,200 x g for 10 minutes at 4°C, after which 100 µl aliquots of cell-free supernatants were stained with 1 µM SYTOX Green dye (Life Technologies) for 10 minutes at RT. Fluorescence was subsequently measured using a BioTek Synergy 2 fluorometric plate reader (NorthStar Scientific Ltd, Sandy, UK) with excitation and emission set at 485 and 528 nm respectively. Data derived from experiments performed in quadruplicate are presented as arbitrary fluorescence units (AFU). The average AFU values of vehicle treated controls were subtracted from PMA-stimulated neutrophils.

### Intracellular cytokine staining

200 µl aliquots of heparinised whole blood were treated for 1 hour with 10 µM indomethacin prior to a 30 minute incubation with 1,500 or 10,000 pg/ml PGE_2_ or vehicle control. Post-treatment, samples were stimulated for 4 hours (37°C/5%CO_2_) with 100 ng/ml LPS in the presence of 3 µg/ml brefeldin A (Merck Life Science UK Limited), after which samples were stained on ice for 30 minutes with combinations of the following mouse anti-human monoclonal antibodies or their concentration-matched isotype controls: 7.5 μg/ml R-phycoerythrin (PE)-Cyanine 7 (PE-Cy7)-labelled CD3 (clone UCHT1; ThermoFisher Scientific, London, UK), 2.5 μg/ml fluorescein isothiocyanate (FITC)-labelled CD14 (clone TUK4; Agilent Technologies, Cheshire, UK), 10 μg/ml FITC-labelled CD19 (clone HIB19; ThermoFisher Scientific) or 1 μg/ml PE-labelled CD56 (clone AF12-7H3; Miltenyi BioTec).

Post-staining, red blood cells were lysed (BD PharmLyse, BD Biosciences), and samples fixed for 15 minutes at RT in 100 µl of fixation medium (Medium A, ThermoFisher Scientific). Following a single wash in PBS (250 x g, 5 minutes, 4°C), samples were incubated for 30 minutes at RT in 100 µl of permeabilisation medium (Medium B, ThermoFisher Scientific) with 5 µg/ml of a PE-labelled mouse anti-human TNF-α antibody (Clone Mab11, BioLegend), 25 µg/ml of a FITC-labelled mouse anti-human TNF-α antibody (Clone Mab11, ThermoFisher Scientific) or their concentration-matched isotype controls. Following a single wash in PBS (250 x g, 5 minutes, 4°C), samples were analysed on a BD FACSVia™ flow cytometer and data examined using CFlow software. TNF-α production was measured as percentage TNF-α positive cells and median fluorescent intensity (MedFI). MedFI values of isotype controls were subtracted from those obtained with test antibodies.

### Measurement of COX-2 protein expression

Following a 1 hour treatment with 1 μM cyclosporin H (CsH, Abcam, Cambridge, UK), 10 µM PD98059 (Cell Signalling Technology, London, UK), 10 µM SB98059 (Cell Signalling Technology), 50 µM LY294002 (Cell Signalling Technology), vehicle control or CM, freshly isolated neutrophils or monocytes (3×10^6^ in CM) were stimulated with 40 or 100 μg/ml mtDAMPs for 3 hours (37°C/5% CO_2_). Post-stimulation, cells were pelleted (1,500 × *g*, 2 min, 4°C), supernatants discarded and cell lysates prepared by the addition of 50 µl hot sodium dodecyl sulphate (SDS) sample buffer (4% SDS (v/v), 0.1 M dithiothreitol, 20% glycerol (v/v), 0.0625 M Tris–HCL and 0.004% bromophenol blue (w/v)). Proteins were separated on 10% SDS-polyacrylamide gels and transferred to polyvinylidene difluoride membranes (Bio-Rad, Hertfordshire, UK). Following a 1 hour incubation at RT with 5% BSA in Tris buffered saline (TBS; 200 mM Tris (pH 7.5), 1.5 M NaCl) containing 0.1% Tween-20 (TBST), blots were probed overnight at 4°C with a rabbit anti-human COX-2 antibody (Clone D5H5; diluted 1:1000 in TBST containing 2.5% BSA; Cell Signalling Technology). Post-incubation, blots were washed in TBST and incubated for 1 hour at RT with a goat anti-rabbit secondary antibody conjugated to horseradish peroxidase (HRP; diluted 1:4000 in TBST; GE Healthcare, Buckinghamshire, UK). HRP activity was detected using enhanced chemiluminescence (Bio-Rad). To confirm equal loading of proteins, blots were probed with a total P38 antibody (Clone D13E1; 1:1000; Cell Signalling Technology). Densitometry analysis was performed using Image J software (National Institutes of Health, Bethesda, MD, USA).

### PGE_2_ production by mtDAMP stimulated neutrophils

Neutrophils (5x10^6^) in CM were treated for 3 hours (37°C/5%CO_2_) with 40 µg/ml mtDAMPs or vehicle control. Post-treatment, cells were pelleted and re-suspended in HBSS^+/+^ prior to a 20 minute culture with 20 uM arachidonic acid (AA). Post-treatment, cells were pelleted (1,500 x g, 2 minutes, 4°C) and cell free supernatants snap frozen in liquid nitrogen. To confirm the role of COX-2 in PGE_2_ production in this system, neutrophils were treated with 10 µM NS398 prior to AA stimulation. To investigate the potential immunomodulatory effects of these supernatants, THP-1 cells (1x10^6^) were treated with 10 µM indomethacin for 1 hour prior to a 30 minute culture (37°C/5%CO_2_) in the abovementioned supernatants. Post-culture, THP-1 cells were stimulated for 2-4 hours with 1 µg/ml LPS, after which cells were pelleted and either supernatants collected for the measurement of TNF-α or pellets re-suspended in 350 µl RLT lysis buffer in preparation for RNA isolation using the commercially available RNeasy isolation kit (Qiagen).

### Efferocytosis assays

Neutrophils isolated from heparinised whole blood were re-suspended at 4x10^6^/ml in RPMI media containing GPS and stained for 45 minutes (37°C/5%CO_2_) with 5 μM Molecular Probes™ CellTracker™ Deep Red Dye (Life Technologies). Stained neutrophils were washed twice in serum free RPMI (500 x g, 5 minutes, RT), re-suspended at 2x10^6^/ml in serum free RPMI. Following overnight incubation (37°C/5%CO_2_), a flow cytometric assessment of neutrophil apoptosis was undertaken using a BD Pharmingen™ FITC Annexin V apoptosis detection kit (BD Biosciences).

Efferocytosis assays were performed by culturing apoptotic neutrophils with MDMs at a ratio of 4:1 for 2 hours (37°C, 5% CO_2_). MDMs treated for 30 minutes with 5 µg/ml of the actin polymerisation inhibitor cytochalasin D (Cyt D, Merck Life Science UK Limited) prior to neutrophil co-culture served as a negative control to account for the adherence of neutrophils to MDMs. An untreated control sample that comprised of MDMs alone was included in all assays to determine autofluorescence of the cells. Following a 2 hour co-culture, media was removed and cells washed twice with ice cold PBS. MDMs were detached from plates via incubation in 1X dissociation buffer (10 minutes, 37°C/5%CO_2_; TrypLE Express, Gibco, ThermoFisher Scientific) and cells washed once in FACS buffer (PBS/2% BSA; 500 x g, 5mins). Cells were transferred to FACS tubes and analysed immediately by flow cytometry on a MACSQuant Analyzer 10 flow cytometer (Miltenyi BioTec) and data examined using FlowJo software (BD Biosciences). Individual samples of MDMs and apoptotic neutrophils were used to set gates for their respective populations on FS/SS plots, with apoptotic neutrophils alone used to set a positive gate to identify MDMs that had engulfed apoptotic neutrophils (**Supplementary Figure 3**). A total of 10,000 MDMs were analysed and the percentage of APC^+^ MDMs recorded. MDMs pre-treated with Cyt D determined a background fluorescence value, which was subtracted from the percentage of APC^+^ MDMs in all samples to generate an efferocytosis index.

### MDM phenotyping

CD215 staining was performed on MDMs treated with 10 µM indomethacin, 10,000 pg/ml PGE_2_ or vehicle control, and 100 ng/ml LPS. Post-treatment, MDMs were washed twice with ice cold PBS and detached from plates via incubation in 1X dissociation buffer (10 minutes, 37°C/5%CO_2_; TrypLE Express). Cells were washed once with FACS buffer (PBS/2% BSA; 500 x g, 5mins, 4^0^C) and incubated at RT for 30 minutes in PBS supplemented with 10% human serum. After a single wash in FACS buffer (500 x g, 5mins, 4^0^C), MDMs were stained for 30 minutes on ice with 5 µg/ml mouse anti-human CD215 (Clone JM7A4, BioLegend) or its concentration-matched isotype control. Post-incubation, samples were washed once in FACS buffer (500 x g, 5mins, 4^0^C), fixed for 10 minutes at RT in 4% paraformaldehyde (PFA) and washed once in FACS buffer (500 x g, 5mins, 4^0^C) in preparation for flow cytometric analysis. 10,000 MDMs were analysed on a MACSQuant Analyzer 10 flow cytometer and data examined using FlowJo software. Data are presented as MFI of CD215 after subtracting background fluorescence of isotype stained controls.

To study SIRP-α expression, MDMs, treated for 1 hour with 10,000 pg/ml PGE_2_ or vehicle control, were fixed for 10 minutes at RT in 4% PFA, washed once in FACS buffer (500 x g, 5mins) and stored overnight at 4^0^C prior to staining. MDMs were stained on ice for 30 minutes with 2.5 µg/ml mouse anti-human SIRP-α FITC (Clone 15-414, Invitrogen) or its concentration-matched isotype control, after which samples were washed once in FACS buffer (500 x g, 5mins, 4°C) and analysed on a MACSQuant Analyzer 10 flow cytometer. Data were analysed using FlowJo software and are presented as MFI of 10,000 MDMs after subtracting the background fluorescence of isotype stained controls.

### Real-time quantitative polymerase chain reaction

For the comparison of gene expression profiles between neutrophils and PBMCs isolated from HC and trauma patients, total RNA was extracted from cell pellets (5x10^6^) re-suspended in 1 ml of TRIzol reagent according to manufacturer’s instructions (Life Technologies). RNA was re-suspended in 20 µl of RNase-free water, heated at 55°C for 10 minutes and its concentration quantified using a NanoDrop 2000 (ThermoFisher Scientific). The effect of mtDAMP treatment on COX-2 gene expression was examined in freshly isolated neutrophils (5x10^6^) and PBMCs (4x10^6^) stimulated for 0, 30, 60 or 120 minutes with 40 µg/ml mtDAMPs. To study the effect of PGE_2_ exposure on TNF-α and IL-15 gene transcription in monocytes, freshly isolated purified monocytes (5x10^5^) from HC were pre-treated with 10 µM indomethacin for 1 hour, followed by a 30 minute culture with 1,500 pg/ml PGE_2_ or vehicle control and a 2 hour stimulation with 100 ng/ml LPS. Post-treatments, neutrophils, PBMCs and monocytes were lysed in RLT buffer (Qiagen) and total RNA extracted using an RNeasy Mini kit according to manufacturer’s instructions (Qiagen). Both extraction protocols isolated RNA with typical A260/A280 ratios of 1.8-2.0, which was deemed suitable for analysis in downstream RT-qPCR experiments.

For RT-qPCR, mRNA levels of COX-2, cPLA_2_, mPEGS-1, EP2, EP4, TNF-α and IL-15 were determined, relative to the housekeeping gene 18S, using the iTaq^™^ Universal SYBR^®^ Green One-step kit mastermix (Bio-Rad) and gene-specific primers (**Supplementary Table 1**). All reactions had a total volume of 5 µl, containing 5 ng of RNA, and were performed in triplicate. For each RT-qPCR performed, a non-template control comprising of only iTaq^™^ Universal SYBR^®^ Green One-step mastermix and gene-specific primers was included to ensure no contamination of PCR reagents. Data was acquired using a Bio-Rad sfx cycler (Bio-Rad) and analysed by the 2^-ΔΔCt^ method using Bio-Rad CFX manager software (Bio-Rad).

### Statistical analyses

Statistical analyses were performed using GraphPad Prism software version 10 (GraphPad Software Ltd, USA). Data distributions were examined using the Kolmogorov-Smirnov and Shapiro Wilk normality tests. For data that followed a normal distribution, paired and unpaired student *t*-tests, and a one-way or repeated measures ANOVA with a Bonferroni’s or Dunnett’s multiple comparison *post hoc* test were performed. For non-normally distributed data, a Wilcoxon matched-pairs signed rank test, a Mann–Whitney *U* test, a Friedman test with Dunn’s multiple comparison *post hoc* test or a Kruskal-Wallis test with Dunn’s multiple comparison *post hoc* test were performed. Relationships between continuous variables were assessed using a Pearson’s or Spearman’s correlation. The threshold for statistical significance was set at p ≤ 0.05.

## Results

### Patient demographics

A total of 95 traumatically-injured patients (86 male, 9 female) were included in this study (**Table 1**). Patients had a mean age of 41 years (range 19-95 years), and a mean ISS of 24 (range 9-66). Road traffic collisions (58%) were the predominant mechanism of injury, with the mean time of pre-hospital blood sampling 41 minutes post-injury (range 14-110 minutes) (**Table 1**).

**Table 1:** Patient demographics.

Characteristic	Patients (n=95)
Age, years (range)	41 (19-95)
Gender, (M:F)	86:9
Time to pre-hospital sample, minutes post-injury (range)	41 (14-110)
ISS (range)^#^	24 (9-66)
Admission GCS score (range)	11 (3-15)
Mechanism of injury *Fall, n (%)* *A/P, n (%)* *Blunt, n (%)* *RTC, n (%)*	11 (12) 25 (26) 4 (4) 55 (58)
ICU-free days (range)	20 (0-30)
Hospital-free days (range)	9 (0-29)
Mortality, n (%)	13 (14)

Data are expressed as mean (range) unless otherwise stated. ^@^Pre-hospital blood samples from 92 patients were analysed in this study. ^#^Data for ISS was available for 91 patients. A/P, Assault/Penetrating; GCS, Glasgow coma scale; ICU, Intensive care unit; ISS, Injury severity score; RTC, Road traffic collision.

### Evidence of impaired innate immune responses in the ultra-early and acute post-injury phase

**Figure 1:**
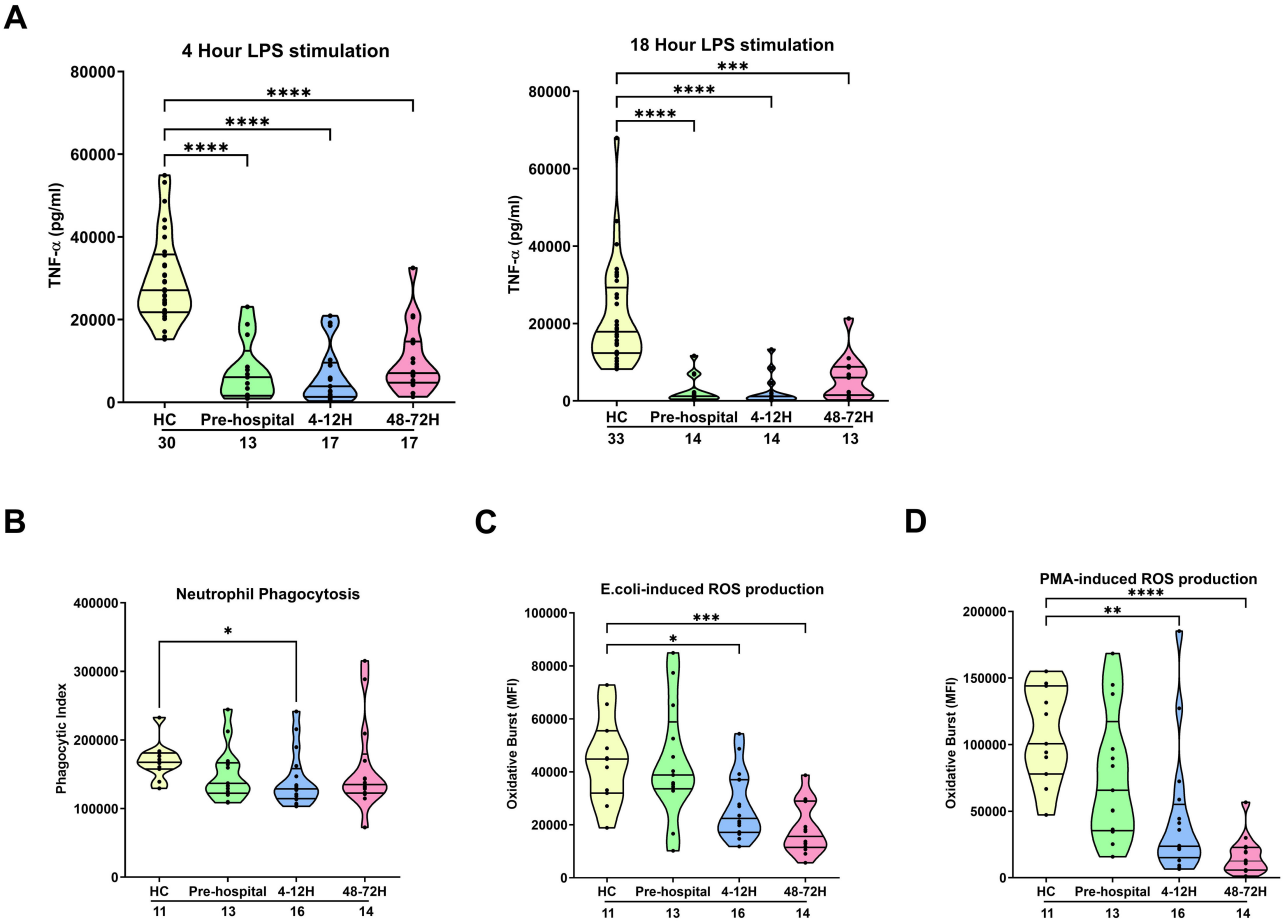
Effect of traumatic injury on *ex vivo* innate immune responses. **(A)** Tumour necrosis factor-alpha (TNF-α) concentrations measured in cell free supernatants obtained from whole blood samples of healthy controls (HC) and trauma patients at 3 post-injury time-points (pre-hospital, 4-12 and 48-72 hours) following a 4 (left panel) or 18 (right panel) hour *ex vivo* stimulation with 100 ng/ml LPS. **(B-D)** Neutrophil phagocytosis of opsonised *E.coli* **(B)** and neutrophil reactive oxygen species production following *E.coli* **(C)** or PMA **(D)** stimulation. ROS data are presented as mean fluorescence intensity (MFI). The number of samples analysed are indicated below each time-point. *p<0.05, **p<0.01, ***p<0.001, ****p<0.0001 Vs HC. *E.coli*, *Escherichia coli*; PMA, phorbol 12-myristate 13-acetate; ROS, reactive oxygen species.

Following a 4 or 18 hour *ex vivo* stimulation with 100 ng/ml LPS, we detected significantly lower concentrations of TNF-α in supernatants of whole blood samples obtained from trauma patients in the pre-hospital setting as well as 4-12 and 48-72 hours post-injury when compared to HC (p<0.0001; **Figure 1A**). This post-trauma induction of endotoxin tolerance was observed in patients that had sustained either moderate (ISS 8-15) or severe (ISS ≥16) injuries, with no significant differences detected in TNF-α production between the two patient subgroups (**Supplementary Table 2**). By 48-72 hours post-injury, we observed no significant difference in LPS-induced cytokine production between monocytes isolated from HC and those from patients with an ISS of 8-15 following an 18 hour LPS stimulation (**Supplementary Table 2**). At none of our three study time-points did LPS-induced TNF-α production correlate with ISS (data not shown).

*Ex vivo* analysis of neutrophil anti-microbial functions revealed a significant trauma-induced impairment in neutrophil phagocytosis of opsonised *E.coli* at our 4-12 hour post-injury sampling time-point (p<0.05; **Figure 1B**) and a significant reduction in ROS generation triggered by *E.coli* and PMA stimulation 4-12 (p<0.05) and 48-72 (p<0.01) hours post-injury (**Figures 1C, D**). At none of our sampling time-points did we find a relationship between either neutrophil ROS production or phagocytic activity and a patients ISS (data not shown).

### Impact of traumatic injury on circulating levels of PGE_2_, albumin and markers of cellular damage

**Figure 2:**
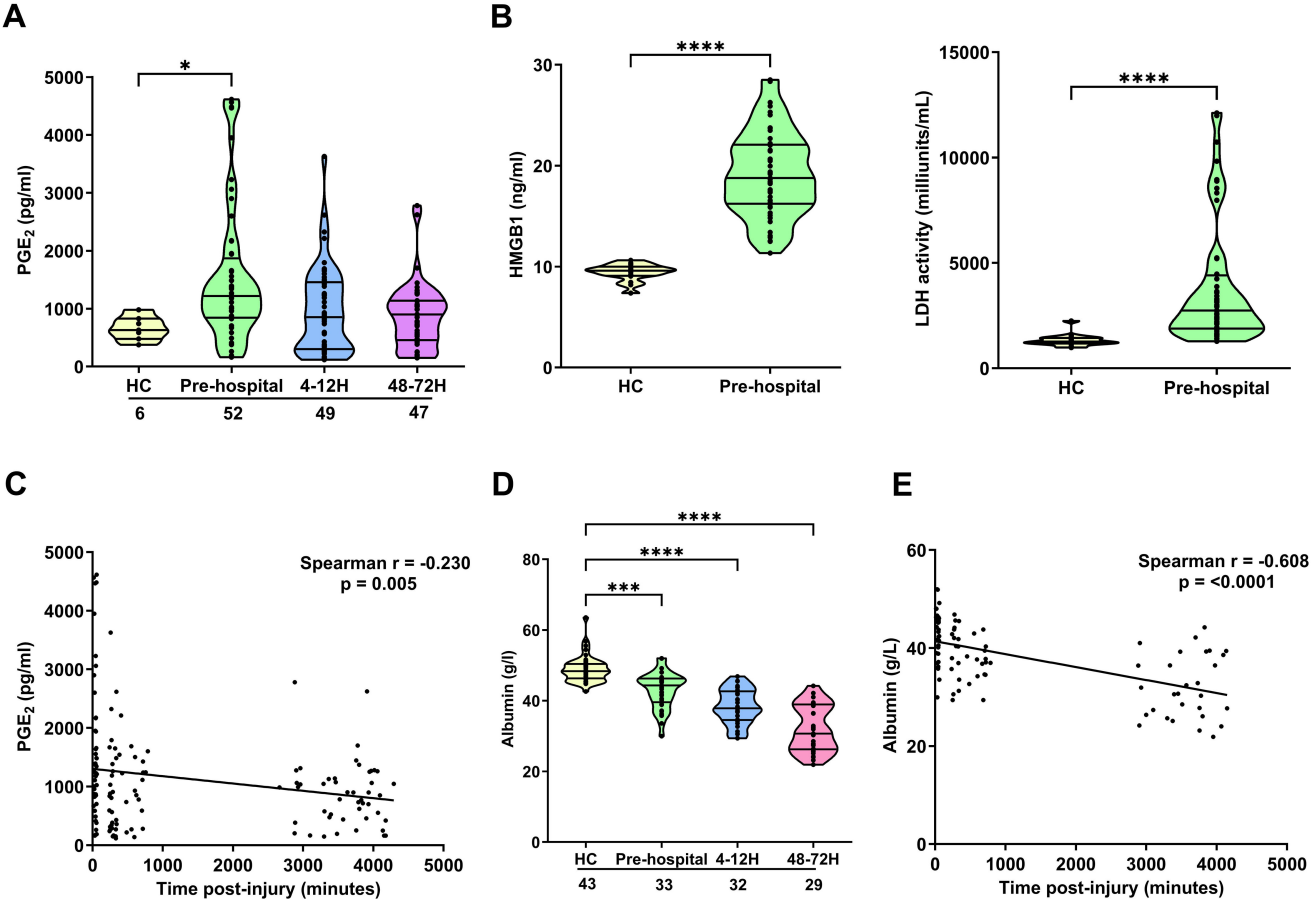
Impact of traumatic injury on circulating concentrations of PGE_2_, albumin and markers of tissue damage. **(A)** Prostaglandin E_2_ (PGE_2_) concentrations measured in serum samples collected from healthy controls (HC) and trauma patients at 3 post-injury time-points (pre-hospital, 4-12 and 48-72 hours). **(B)** HMGB-1 levels (left panel; HC, n=16, pre-hospital, n=52) and LDH activity (right panel; HC, n=16, pre-hospital, n=56) in serum samples obtained from HC and trauma patients in the pre-hospital setting. **(C)** Spearman’s correlation examining the relationship between serum PGE_2_ concentrations and time of blood sample acquisition post-injury (n=143). **(D)** Albumin concentrations in serum samples of HC and trauma patients at 3 post-injury time-points (pre-hospital, 4-12 and 48-72 hours). **(E)** Spearman’s correlation depicting the relationship between serum albumin concentrations and time of blood sample acquisition post-injury (n=86). The number of samples analysed are indicated below each time-point. *p<0.05, ***p<0.001, ****p<0.0001 Vs HC. HMGB-1, High Mobility Group Box-1; LDH, lactate dehydrogenase.

Compared to values obtained for HC, we detected significantly higher concentrations of PGE_2_ in blood samples acquired from trauma patients within 1 hour of injury (p<0.05; **Figure 2A**). This ultra-early increase in PGE_2_ levels was accompanied by significantly elevated circulating concentrations of the DAMP HMGB-1 and significantly higher serum LDH activity (p<0.0001; **Figure 2B**). An assessment of PGE_2_ concentrations across our three study time-points revealed a significant negative association between PGE_2_ levels and the time of sample acquisition post-injury (**Figure 2C**). As shown in **Supplementary Figure 4A**, we detected no significant differences in circulating PGE_2_ levels between male and female trauma patients, nor did we find any relationship between PGE_2_ concentrations ≤1 hour post-injury and a patients ISS (r(n=51)=0.025, p=0.860).

Albumin binds to, and counteracts, the immune suppressive effects of PGE_2_ (42, 43). At all three of our post-injury time-points, we detected a significant trauma-induced reduction in serum albumin concentrations (p<0.001; **Figure 2D**). An assessment of the relationship between albumin levels and time of sample acquisition post-injury revealed a significant negative association between these two variables (**Figure 2E**). No significant differences in serum albumin concentrations were detected between male and female trauma patients at any of our sampling time-points (**Supplementary Figure 4B**).

In a small number of patients (n=8), paired measurements of PGE_2_ concentrations and ROS generation by E.coli and PMA stimulated neutrophils were obtained at the 48-72 hour sampling time-point (**Supplementary Table 3**). We found a significant negative association between circulating PGE_2_ levels and PMA-induced ROS production (r(n=8)=-0.759, p=0.03). Although not reaching statistical significance, a trend for an inverse relationship between PGE_2_ concentrations and ROS generation by *E.coli* challenged neutrophils was also observed (r(n=8)=-0.693, p=0.06).

### Effect of traumatic injury on the expression of enzymes and receptors involved in PGE_2_ synthesis and signalling

To investigate whether trauma altered the expression of genes involved in the synthesis and detection of PGE_2_ by leukocytes, we compared the mRNA levels of enzymes and receptors involved in PGE_2_ production and signalling in neutrophils and PBMCs isolated from trauma patients and HC.

**Figure 3:**
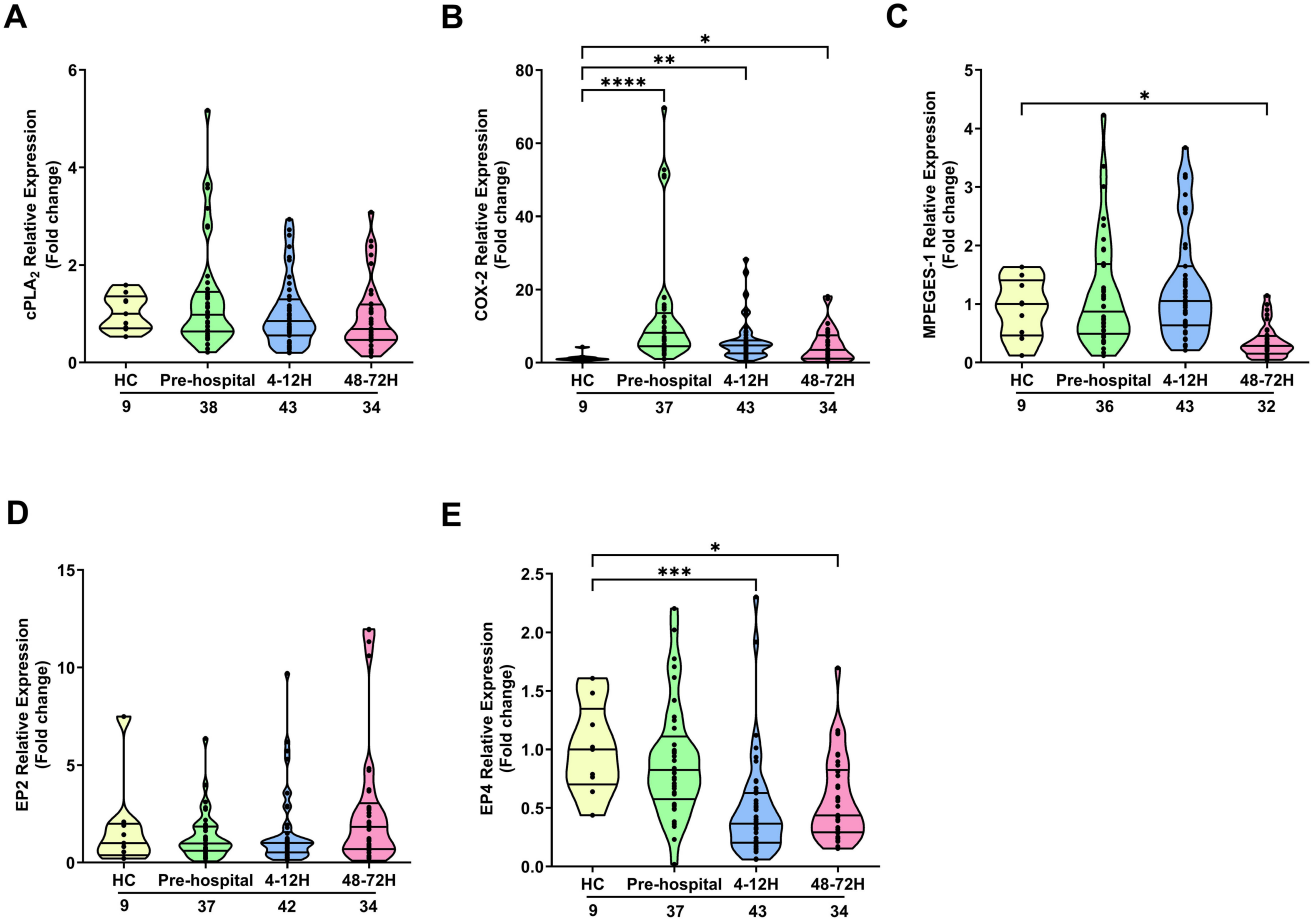
Effect of traumatic injury on the expression of enzymes and receptors involved in prostaglandin E_2_ synthesis and signalling in neutrophils. **(A-E)** Comparison of cPLA_2_ **(A)**, COX-2 **(B)**, mPGES-1 **(C)**, EP2 **(D)** and EP4 **(E)** gene expression in freshly isolated neutrophils of healthy controls (HC) and trauma patients at 3 post-injury time-points (pre-hospital, 4-12 and 48-72 hours). The number of samples analysed are indicated below each time-point. *p<0.05, **p<0.01, ***p<0.001, ****p<0.0001 Vs HC. COX-2, Cyclooxygenase-2; cPLA_2_, cytosolic phospholipase A2; EP2, E prostanoid receptor 2; EP4, E prostanoid receptor 4; mPGES-1, microsomal prostaglandin E synthase-1.

In neutrophils, traumatic injury had no effect upon the mRNA levels of cPLA_2_ (**Figure 3A**). In contrast, at all post-injury sampling time-points, a significant trauma-induced increase in COX-2 gene expression was observed (p<0.05; **Figure 3B**). No differences in mPGES-1 mRNA levels were detected in neutrophils isolated from HC and trauma patients in the pre-hospital setting or 4-12 hours post-injury (**Figure 3C**). However, a significant trauma-induced reduction in mPGES-1 gene expression was observed 48-72 hours post-injury (p<0.05; **Figure 3C**). Examination of EP2 and EP4 expression revealed traumatic injury resulted in a significant reduction in the mRNA levels of EP4 4-12 and 48-72 hours post-injury (p<0.05; **Figures 3D, E**). At all other sampling time-points, EP2 and EP4 gene expression was comparable between neutrophils isolated from HC and trauma patients (**Figures 3D, E**). A comparison of gene expression between neutrophils isolated from male and female patients revealed that females presented with significantly higher EP2 mRNA levels 4-12 hours post-injury (**Supplementary Table 4**). However, as reported for the entire patient cohort, no significant difference in EP2 expression was detected between neutrophils from male or female patients at this sampling time-point when compared to HC (Relative expression; HC, 1.74 ± 0.75, Males, 1.25 ± 0.23, Females, 19.43 ± 15.33). No gender differences in the expression of other PGE2 associated genes were found (**Supplementary Table 4**).

**Figure 4:**
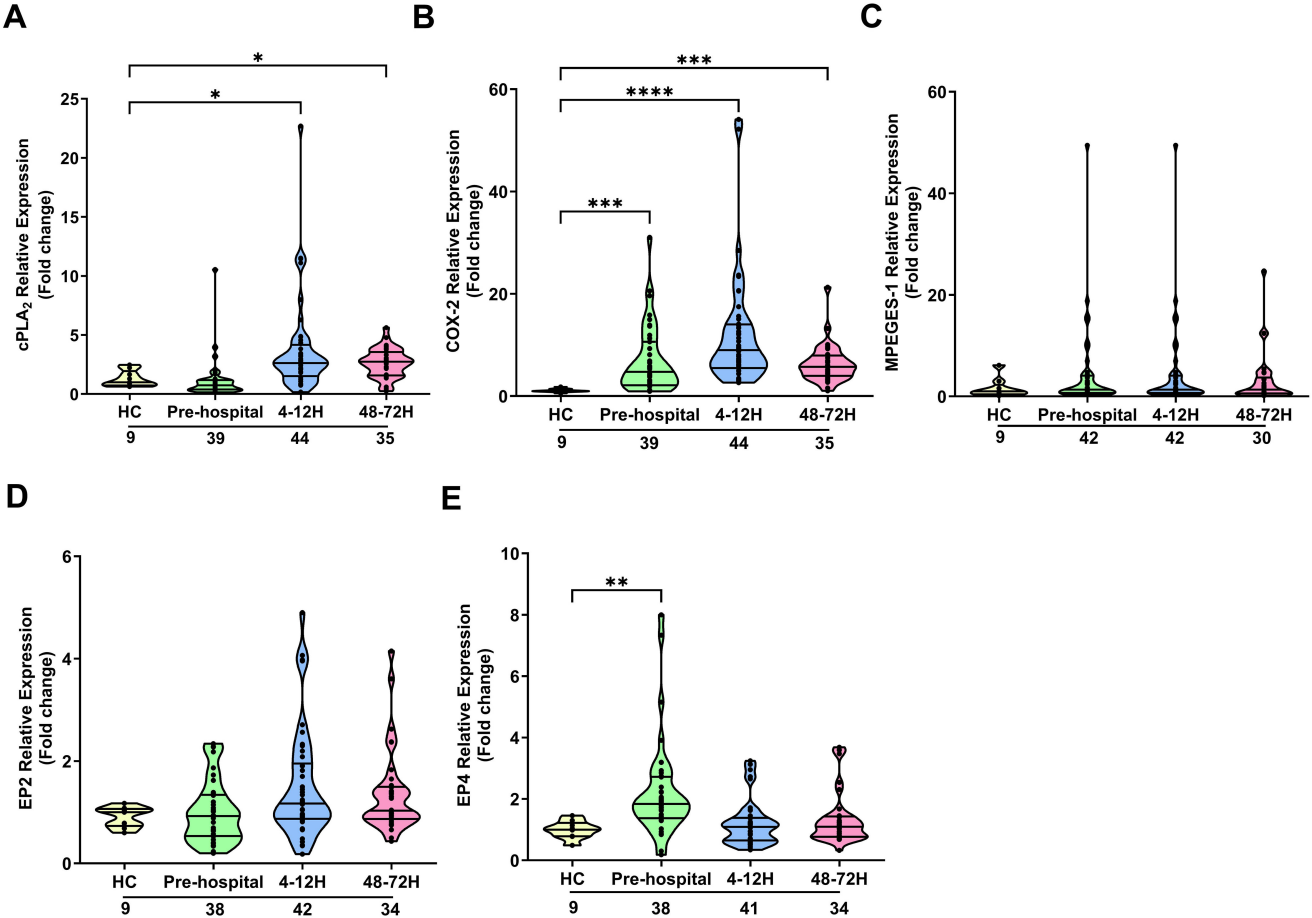
Effect of traumatic injury on the expression of enzymes and receptors involved in prostaglandin E_2_ synthesis and signalling in peripheral blood mononuclear cells. **(A-E)** Comparison of cPLA_2_ **(A)**, COX-2 **(B)**, mPGES-1 **(C)**, EP2 **(D)** and EP4 **(E)** gene expression in freshly isolated PBMCs of healthy controls (HC) and trauma patients at 3 post-injury time-points (pre-hospital, 4-12 and 48-72 hours). The number of samples analysed are indicated below each time-point. *p<0.05, **p<0.01, ***p<0.001, ****p<0.0001 Vs HC. COX-2, Cyclooxygenase-2; cPLA_2_, cytosolic phospholipase A2; EP2, E prostanoid receptor 2; EP4, E prostanoid receptor 4; mPGES-1, microsomal prostaglandin E synthase-1.

In contrast to neutrophils, PBMCs isolated from trauma patients 4-12 and 48-72 hours post-injury exhibited significantly elevated cPLA_2_ mRNA expression when compared to HC (p<0.05; **Figure 4A**). A significant trauma-induced increase in COX-2 mRNA levels was observed at all sampling time-points (p<0.001; **Figure 4B**). Traumatic injury had no effect upon the expression by PBMCs of mPGES-1 (**Figure 4C**) or EP2 (**Figure 4D**). Relative to HC, EP4 gene expression showed a significant trauma-induced up-regulation at the pre-hospital sampling time-point (p<0.01; **Figure 4E**), with no significant differences observed at either the 4-12 and 48-72 hour post-injury time-points (**Figure 4E**). A comparison of gene expression between male and female patients revealed significantly higher COX-2 mRNA levels in PBMCs isolated from females 4-12 hours post-injury (**Supplementary Table 4**). When split by gender, COX-2 gene expression in PBMCs obtained from both male and female patients 4-12 hours post-trauma was significantly higher when compared to HC (Relative expression; HC, 1.09 ± 0.12, Males, 11.12 ± 1.80^****^, Females, 18.02 ± 3.39^****^, ^****^p<0.0001 Vs HC). No gender differences in the expression of other PGE2 associated genes were found (**Supplementary Table 4**).

### *In vitro* PGE_2_ treatment suppresses monocyte and neutrophil anti-microbial responses

**Figure 5:**
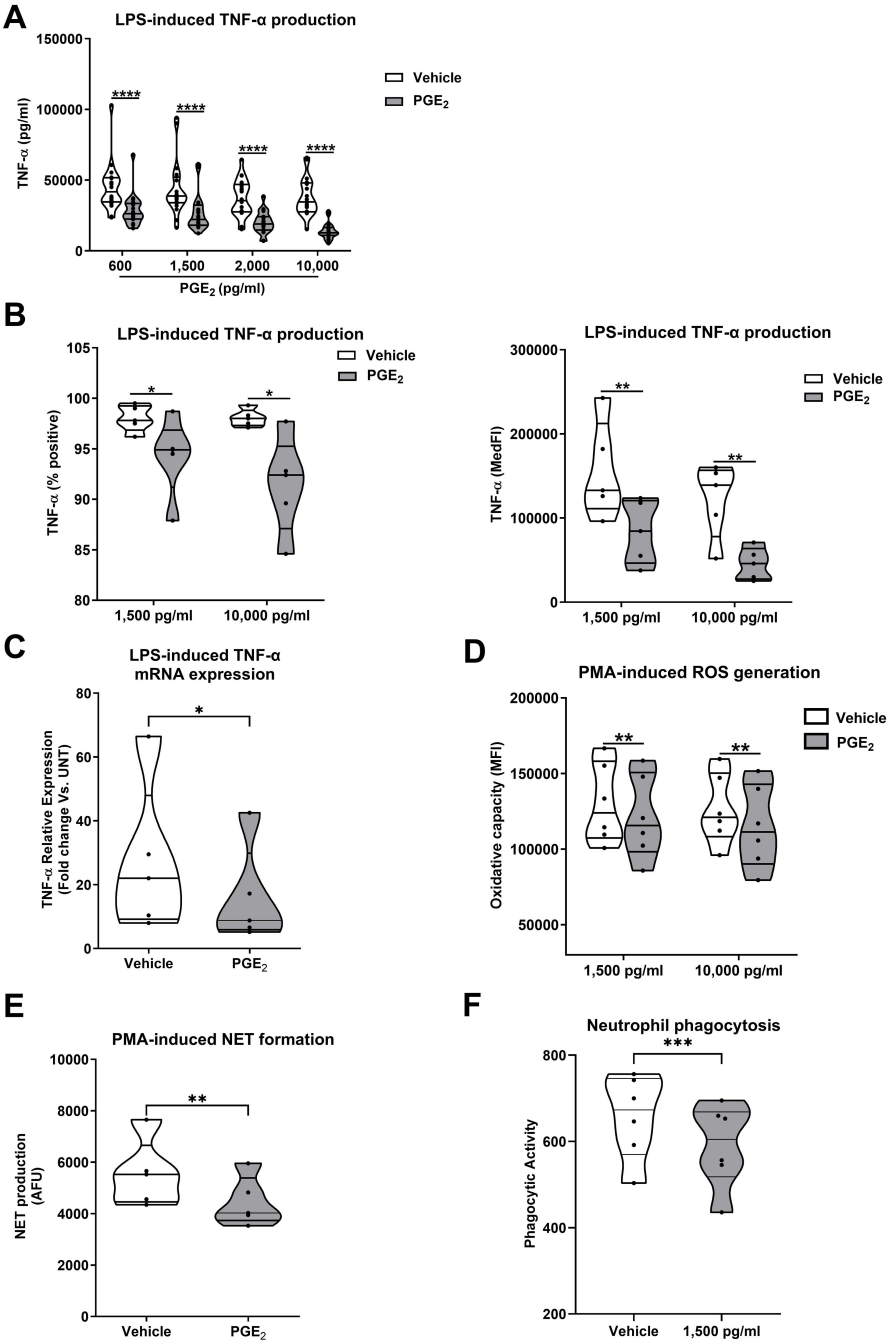
Exogenous prostaglandin E_2_ (PGE_2_) treatment suppresses monocyte and neutrophil anti-microbial responses. **(A)** Tumour necrosis factor-alpha (TNF-α) concentrations in cell free supernatants of 4 hour lipopolysaccharide (LPS) challenged whole blood samples pre-treated with PGE_2_ (600-10,000 pg/ml) or vehicle control (n=20). **(B)** Flow cytometric analysis of TNF-α production by LPS stimulated monocytes pre-treated with 1,500 or 10,000 pg/ml PGE_2_ or vehicle control (n=5). Data are presented as the percentage of TNF-α positive monocytes (left panel) and median fluorescence intensity (MedFI, right panel). **(C)** TNF-α mRNA levels (relative to untreated controls) in 2 hour LPS stimulated primary human monocytes pre-treated with 1,500 pg/ml PGE_2_ or vehicle control (n=5). **(D, E)** PMA-induced ROS production (n=7) **(D)** and NET formation (n=5) **(E)** by neutrophils pre-treated with 1,500 pg/ml PGE_2_ or vehicle control. **(F)** Phagocytic activity of neutrophils pre-treated with 1,500 pg/ml PGE_2_ or vehicle control (n=6). *p<0.05, **p<0.01, ***p<0.001, ****p<0.0001. MFI, Mean fluorescence intensity; NET, neutrophil extracellular traps; PMA, phorbol 12-myristate 13-acetate; ROS, reactive oxygen species.

To investigate the effect of exogenous PGE_2_ treatment on leukocyte cytokine production, we pre-treated whole blood samples from HC for 30 minutes with 4 doses of PGE_2_ (600, 1,500, 2,000 or 10,000 pg/ml) prior to a 4 hour stimulation with 100 ng/ml LPS. At all doses, PGE_2_ pre-treatment significantly reduced LPS-induced TNF-α production (p<0.0001; **Figure 5A**).

Flow cytometry analysis revealed monocytes were the predominant source of TNF-α in LPS challenged whole blood (**Supplementary Figure 5**). When assessed as the percentage of TNF-α positive cells or MedFI values, we observed significantly reduced TNF-α production by monocytes in LPS-challenged whole blood samples that had been pre-treated with 1,500 or 10,000 pg/ml PGE_2_ (p<0.05; **Figure 5B**). This PGE_2_-induced impairment of monocyte TNF-α production was associated with reduced gene transcription, with freshly isolated primary human monocytes pre-treated with 1,500 pg/ml PGE_2_ exhibiting a blunted induction of TNF-α mRNA expression upon LPS challenge (p<0.05; **Figure 5C**).

In addition to monocytes, exposure to PGE_2_ also impaired neutrophil function. Pre-treatment of neutrophils isolated from HC with 1,500 pg/ml and/or 10,000 pg/ml PGE_2_ significantly reduced PMA-induced ROS generation (p<0.01; **Figure 5D**), NET production (p<0.01; **Figure 5E**) and neutrophil phagocytic activity (p<0.001 **Figure 5F**).

### PGE_2_ suppresses TNF-α production by whole blood leukocytes in an EP4 dependent manner

**Figure 6:**
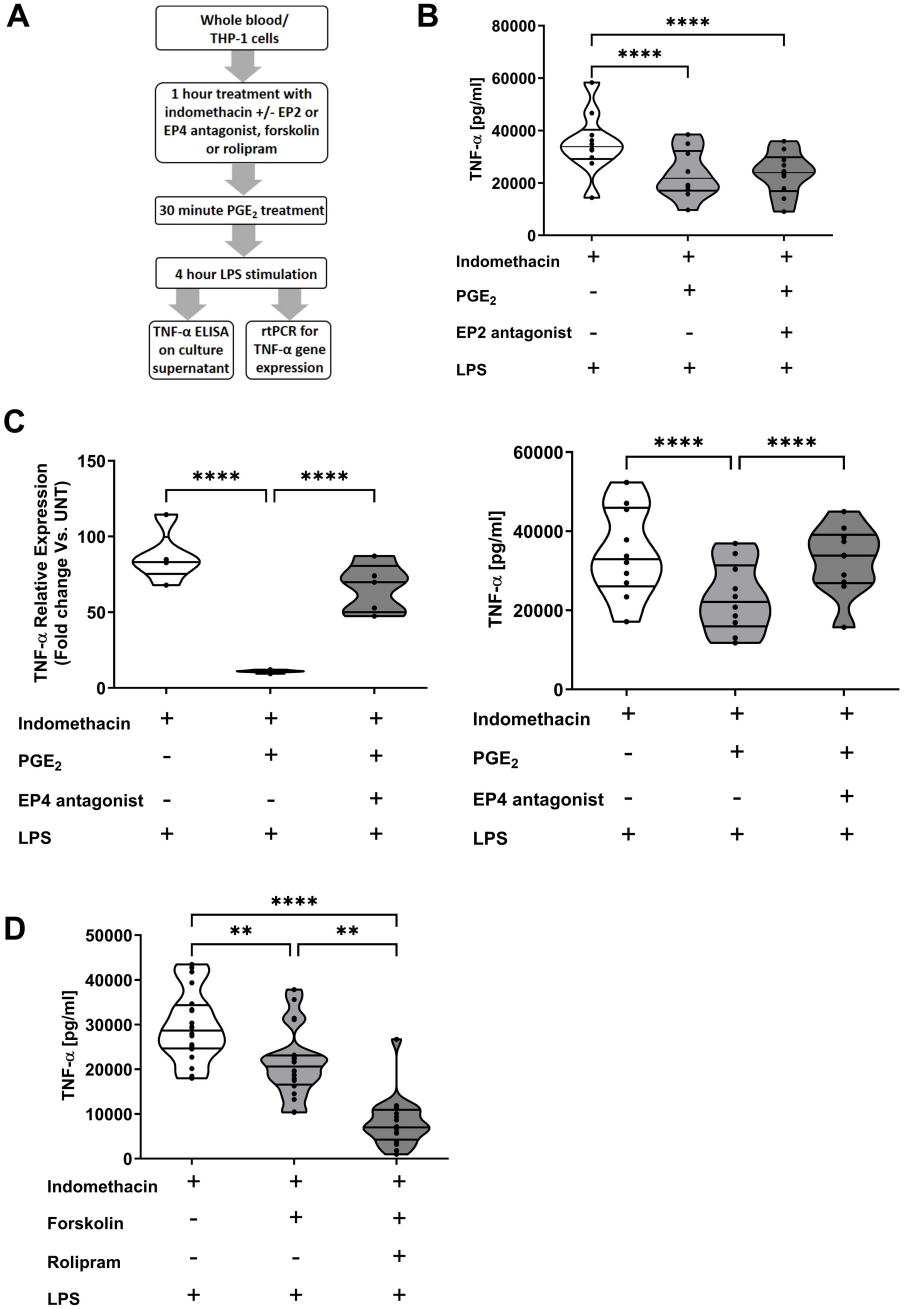
Prostaglandin E_2_ (PGE_2_) suppresses tumour necrosis factor-alpha (TNF-α) production by monocytes in an EP4 and cyclic adenosine monophosphate (cAMP) dependent manner. **(A)** Overview of experimental design of studies investigating the role of the receptors EP2 and EP4, the enzyme protein kinase A and the second messenger cAMP in PGE_2_-induced suppression of TNF-α production by whole blood leukocytes and THP-1 cells. **(B)** Whole blood samples from healthy controls (n=10) were treated for 1 hour with 10 µM of the EP2 receptor antagonist AH6809 prior to a 30 minute incubation with 1,500 pg/ml PGE_2_. Post-treatment, samples were stimulated for 4 hours with 100 ng/ml lipopolysaccharide (LPS), after which TNF-α concentrations in cell free supernatants were measured. **(C)** Left panel; THP-1 cells were pre-treated for 1 hour with 10 µM of the EP4 receptor antagonist L161,982 prior to a 30 minute incubation with 1,500 pg/ml PGE_2_. Post-treatment, THP-1 cells were stimulated for 2 hours with 1 µg/ml LPS, after which TNF-α mRNA expression was measured (n=5). Right panel; Whole blood samples from healthy controls (n=10) were treated for 1 hour with 10 µM of the EP4 receptor antagonist L161,982 prior to a 30 minute incubation with 1,500 pg/ml PGE_2_. Post-treatment, samples were stimulated for 4 hours with 100 ng/ml LPS, after which TNF-α concentrations in cell free supernatants were determined. **(D)** Direct activation of the cAMP generating enzyme adenylate cyclase via a 1 hour pre-treatment of whole blood samples with 100 µM forskolin significantly reduced TNF-α production by leukocytes stimulated for 4 hours with 100 ng/ml LPS. This impairment in TNF-α synthesis was potentiated by co-treatment with 10 µM rolipram, an inhibitor of the cAMP hydrolysing enzyme PDE4 (n=20). **p<0.01, ****p<0.0001.

To determine the signalling pathways involved in the PGE_2_-induced suppression of TNF-α production, whole blood leukocytes or THP-1 cells were treated with the EP2 receptor antagonist AH6809 or the EP4 receptor antagonist L161,982 prior to PGE_2_ and LPS stimulation (**Figure 6A**). AH6809 pre-treatment did not prevent PGE_2_-induced suppression of TNF-α secretion (p<0.0001; **Figure 6B**). In contrast, L161,982 treatment reversed the PGE_2_-induced decrease in LPS stimulated TNF-α gene transcription and secretion that was observed in vehicle pre-treated THP-1 cells and leukocytes respectively (p<0.0001; **Figure 6C**).

Pre-treatment of blood samples with the adenylate cyclase activator forskolin significantly reduced LPS-induced TNF-α production (p<0.01; **Figure 6D**). PDE4 is the enzyme primarily responsible for the hydrolysis of cAMP that is generated by adenylate cyclase. Demonstrating the immune suppressive actions of cAMP, we found that whole blood leukocytes co-treated with forskolin and the PDE4 inhibitor rolipram produced significantly less TNF-α upon LPS stimulation when compared to whole blood samples treated with forskolin alone (p<0.01; **Figure 6D**).

### Effect of PGE_2_ treatment on the function and phenotype of MDMs and primary human monocytes

**Figure 7:**
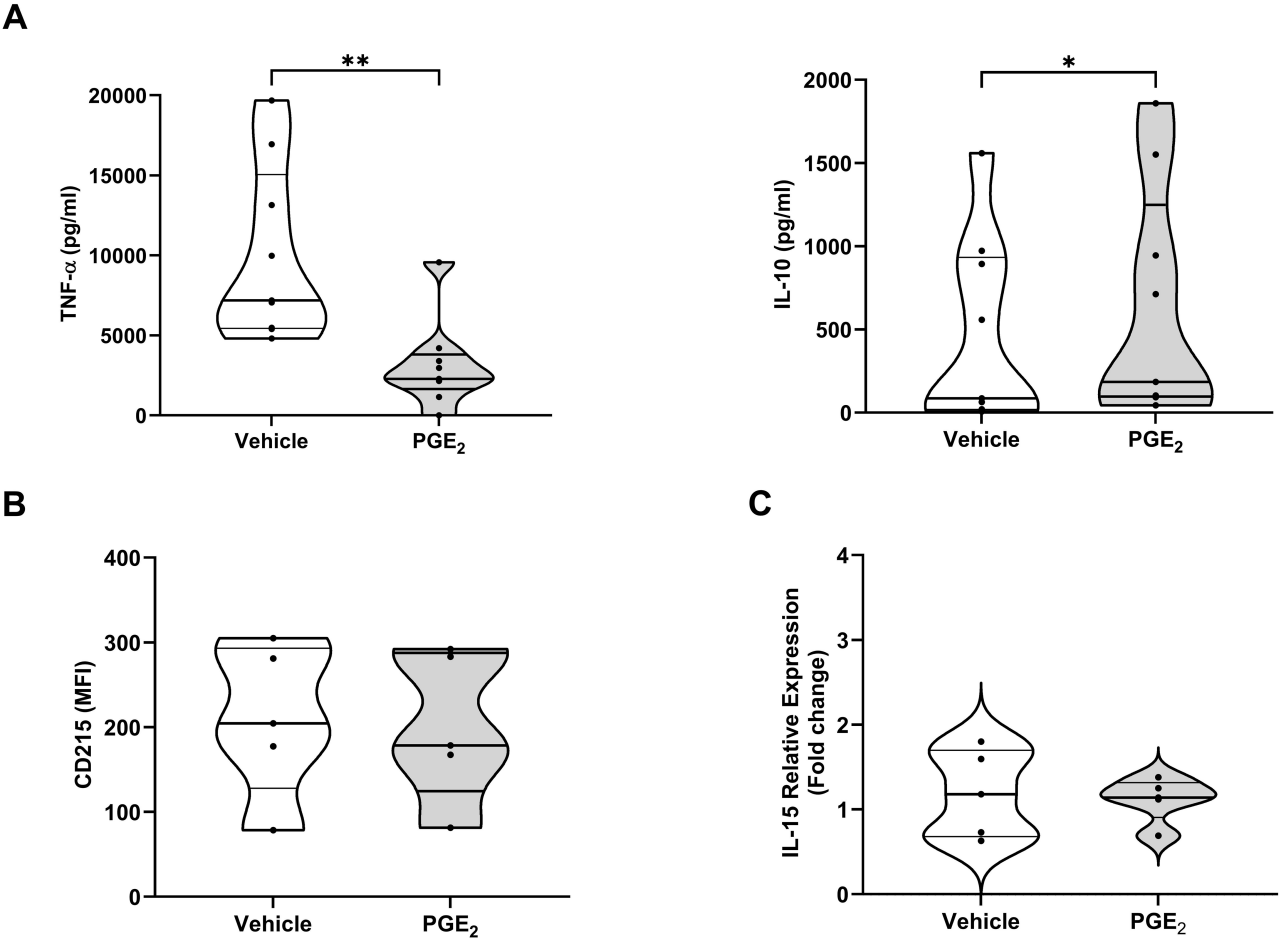
Effect of prostaglandin E2 (PGE_2_) treatment on the function and phenotype of lipopolysaccharide (LPS) stimulated primary human monocytes and monocyte derived macrophages (MDMs). **(A)** Concentrations of tumour necrosis factor-alpha (TNF-α, left panel) and interleukin (IL)-10 (right panel) measured in supernatants derived from 4 hour LPS (100 ng/ml) challenged MDMs pre-treated for 1 hour with 10,000 pg/ml PGE_2_ or vehicle (n=9). **(B)** Expression of CD215 on the surface of 4 hour LPS challenged MDMs pre-treated for 1 hour with 10,000 pg/ml PGE_2_ or vehicle (n=5). **(C)** Comparison of IL-15 mRNA levels in 2 hour LPS challenged primary human monocytes pre-treated for 1 hour with 10,000 pg/ml PGE_2_ or vehicle (n=5). *p<0.05, **p<0.01. MFI, Mean fluorescence intensity.

In line with our data obtained with circulating monocytes (**Figure 5B**), MDMs pre-treated with PGE_2_ secreted significantly lower amounts of TNF-α following LPS stimulation (**Figure 7A**). The profound state of systemic immune suppression that develops following major trauma has been shown to result in reactivation of latent herpes viruses (44–46). Previous studies have reported concentrations of IL-10 are elevated in injured patients who experience viral reactivation (44), whilst IL-15 production by myeloid cells has been shown to counteract herpes viral reactivation (47). Compared to vehicle controls, MDMs pre-treated with 10,000 pg/ml PGE_2_, a concentration detected at sites of active inflammation (41), produced significantly higher amounts of IL-10 in response to LPS challenge (**Figure 7A**). When synthesised, IL-15 is trans-presented by the surface molecule CD215. We detected no difference in the expression of CD215 on the surface of LPS challenged MDMs pre-treated with PGE_2_ or vehicle control (**Figure 7B**). An examination of IL-15 mRNA levels in primary human monocytes found prior exposure to PGE_2_ had no effect upon LPS-induced IL-15 gene transcription (**Figure 7C**).

**Figure 8:**
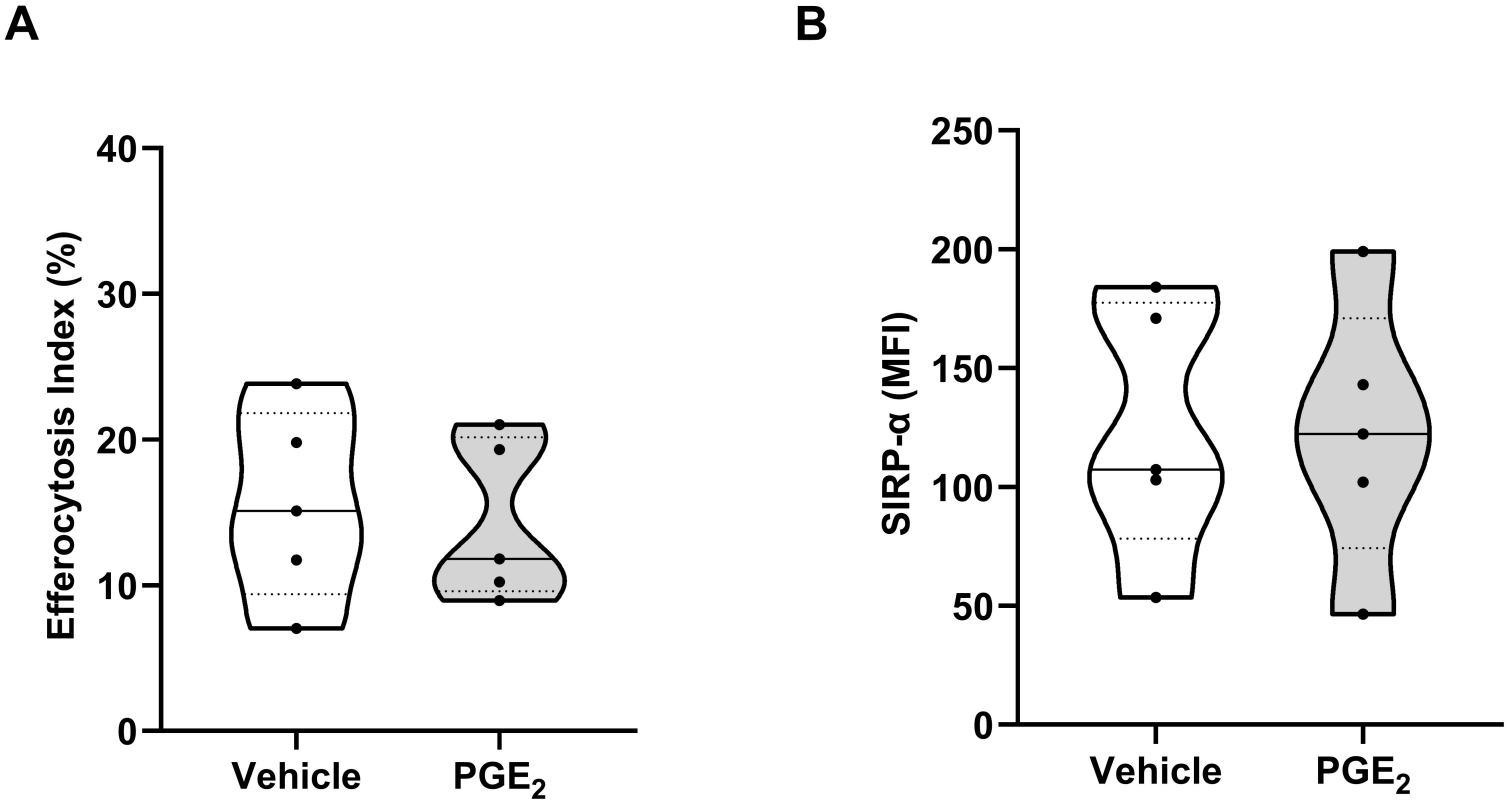
Effect of prostaglandin E2 (PGE_2_) treatment on efferocytosis by monocyte-derived macrophages (MDMs). **(A)** Comparison of efferocytosis index of MDMs pre-treated for 1 hour with 10,000 pg/ml PGE_2_ or vehicle (n=5). **(B)** Expression of signal regulatory protein-alpha (SIRP-α) on the surface of MDMs treated for 1 hour with 10,000 pg/ml PGE_2_ or vehicle (n=5). MFI, Mean fluorescence intensity.

Efferocytosis, the clearance of apoptotic cells by phagocytes, contributes to the resolution of inflammatory responses (48). We found that pre-treatment with PGE_2_ did not affect the ability of MDMs to engulf apoptotic neutrophils (**Figure 8A**) nor did it alter the surface expression of SIRP-α, a negative regulator of efferocytosis (49) (**Figure 8B**).

### MtDAMP stimulation induces COX-2 expression in leukocytes in a formyl peptide receptor-1 and extracellular signal-regulated protein kinase 1/2 dependent manner

Released into the circulation within minutes of injury, mtDAMPs are potent activators of innate immune cells (4, 38, 50). To investigate whether acute exposure to mtDAMPs could induce COX-2 expression in leukocytes, we treated neutrophils, PBMCs and monocytes isolated from HC with mtDAMPs for 30-180 minutes and measured COX-2 gene and/or protein expression.

**Figure 9:**
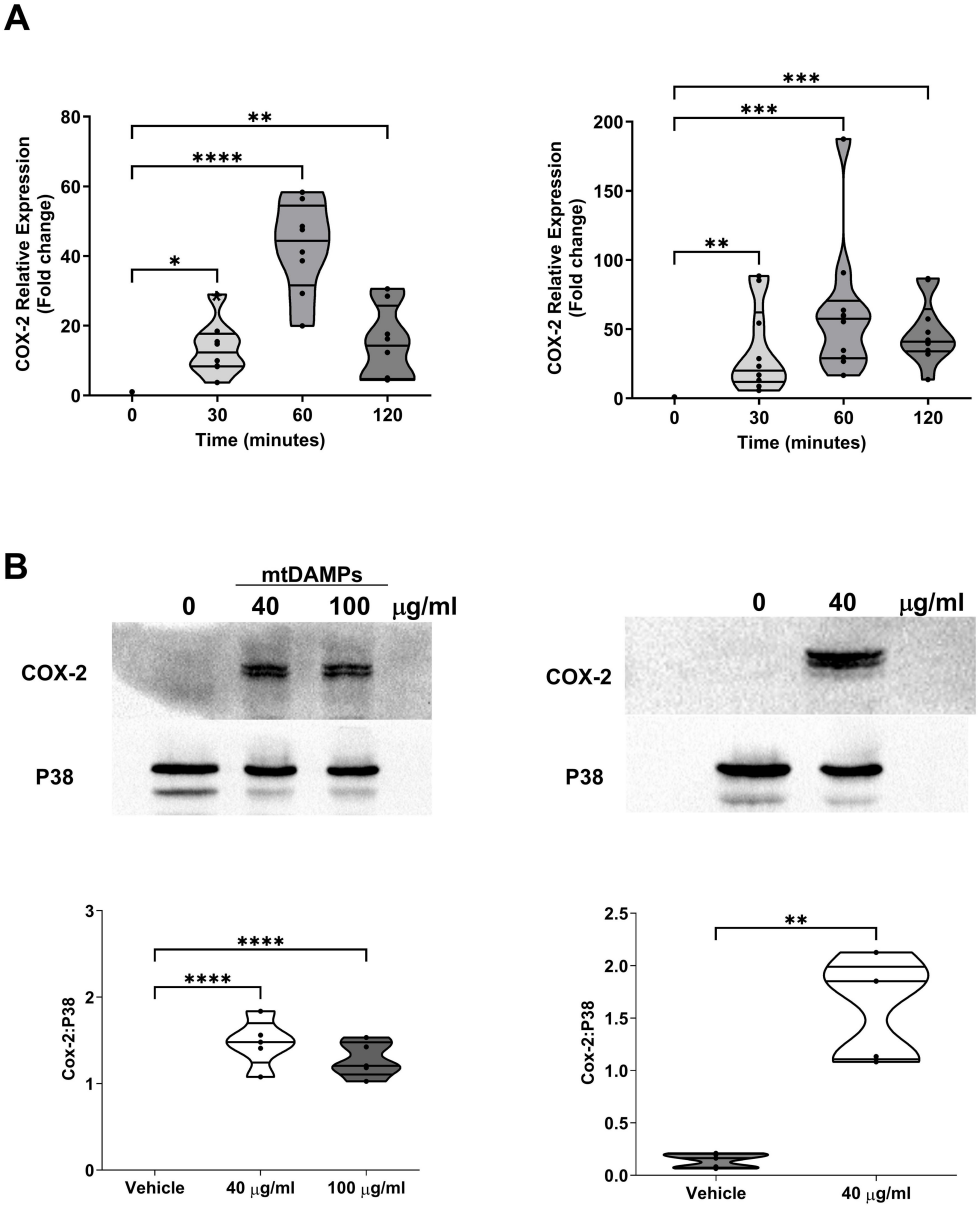
Exposure to mitochondrial-derived damage associated molecular patterns (mtDAMPs) induces cyclooxygenase-2 (COX-2) expression in leukocytes. **(A)** COX-2 gene expression in neutrophils (left panel; n = 8) and PBMCs (right panel; n = 10) isolated from healthy controls (HC) following a 30, 60 and 120 minute *ex vivo* stimulation with 40 µg/ml mtDAMPs. **(B)** COX-2 protein expression in neutrophils (left panel) and monocytes (right panel) of HC following a 3 hour stimulation with 40 and/or 100 µg/ml mtDAMPs. Data are presented as a representative Western blot (top panels) and densitometry analysis (bottom panels) of 5 independent experiments. *p<0.05, **p<0.01, ***p<0.001, ****p<0.0001.

Relative to untreated samples, COX-2 gene expression was significantly higher in neutrophils and PBMCs stimulated for 30, 60 and 120 minutes with 40 µg/ml mtDAMPs (p<0.05; **Figure 9A**). In purified neutrophils and monocytes, a mtDAMP-induced increase in COX-2 protein expression was also observed (p<0.01; **Figure 9B**).

**Figure 10:**
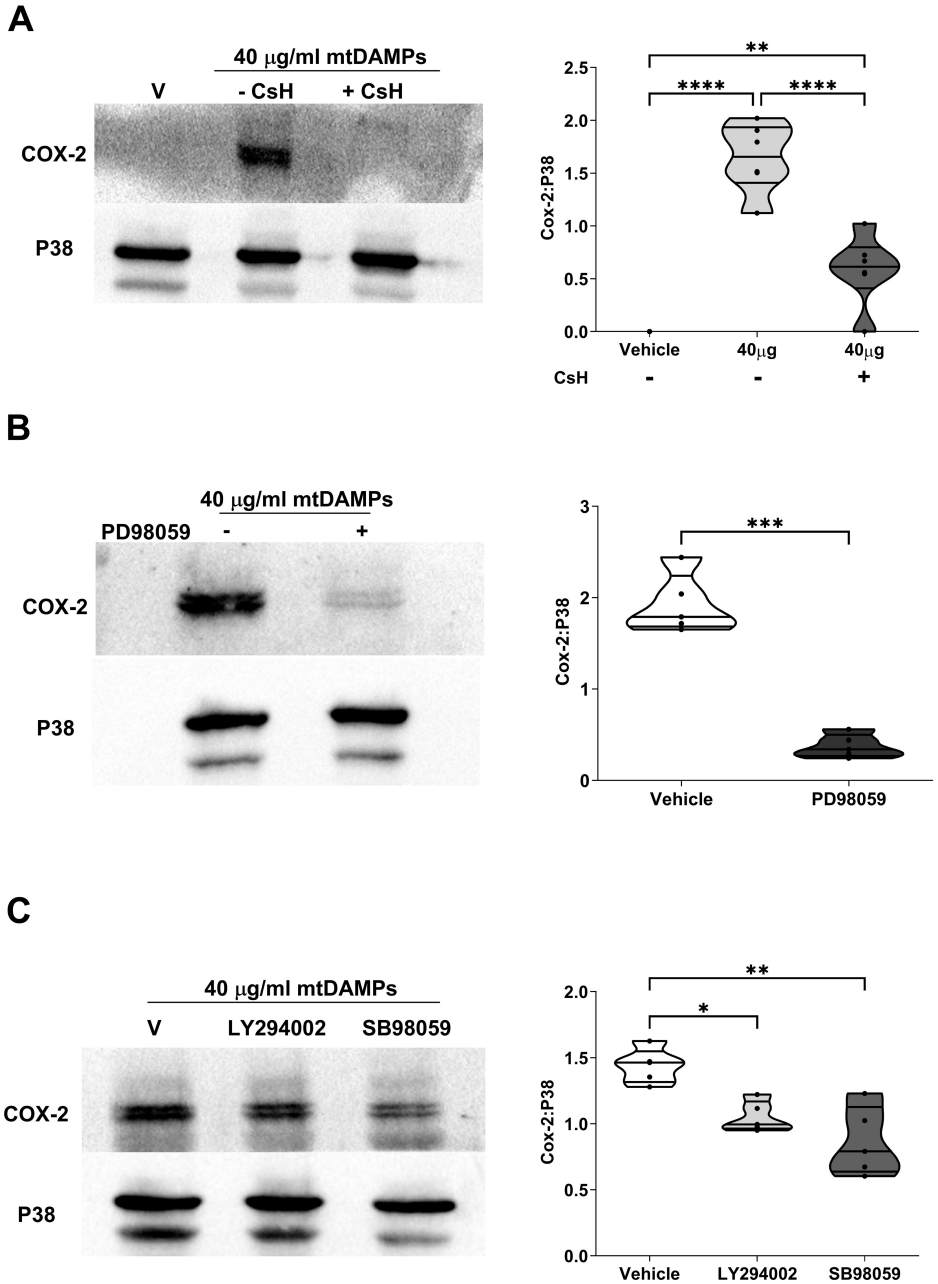
Mitochondrial-derived damage associated molecular pattern (mtDAMP)-induced expression of cyclooxygenase-2 (COX-2) in neutrophils is mediated through formyl peptide receptor-1 (FPR-1) signalling and the activation of extracellular signal-regulated protein kinase 1/2 (ERK 1/2), P38 and Pi3-kinase. **(A-C)** Pre-treatment of neutrophils for 1 hour with 1 µM of the selective FPR-1 antagonist CsH **(A)**, 10 µM of the ERK1/2 inhibitor PD98059 **(B)**, 50 µM of the Pi3-kinase inhibitor LY294002 **(C)** or 10 µM of the P38 inhibitor SB980539 **(C)** significantly reduced the 40 µg/ml mtDAMP-induced upregulation of COX-2 in neutrophils. Data are presented as a representative Western blot (left panels) and densitometry analysis (right panels) of 6 **(A)** and 5 **(B, C)** independent experiments. *p<0.05, **p<0.005, ***p<0.0005 Vs Vehicle or time 0. CsH, cyclosporin H; ERK1/2, extracellular signal-regulated protein kinase 1/2.

Within mtDAMP preparations, N-formylated peptides are potent activators of neutrophils (33). Demonstrating a role for N-formylated peptides in the mtDAMP-induced up-regulation of COX-2 in neutrophils, pre-treatment with the selective FPR-1 antagonist CsH significantly reduced the mtDAMP-induced increase in COX-2 protein expression observed in neutrophils treated with vehicle control (p<0.01; **Figure 10A**). Downstream of FPR-1, a role for MAPKs and Pi3-kinase signalling in mediating the mtDAMP-induced increase in COX-2 expression was demonstrated by experiments that showed that pre-treatment with the ERK1/2 inhibitor PD98059, the P38 inhibitor SB98059 or the phosphoinositide 3-kinase (Pi3-kinase) inhibitor LY2940002 significantly reduced the up-regulation in COX-2 protein expression that was observed in vehicle treated neutrophils stimulated with 40 µg/ml mtDAMPs (p<0.05; **Figures 10B, C**).

### Induction of COX-2 expression by mtDAMPs in neutrophils results in secretion of PGE_2_.

**Figure 11:**
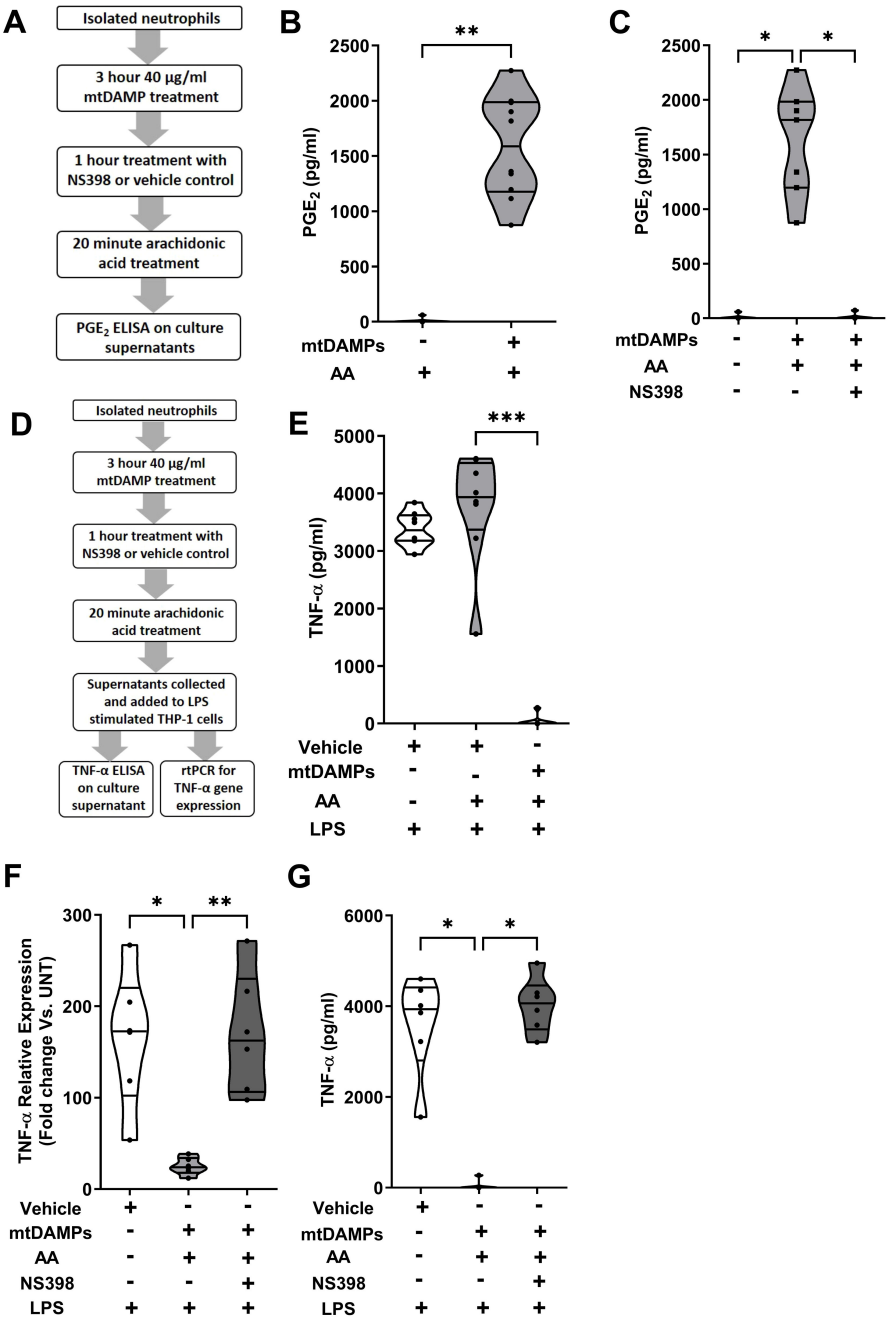
Induction of cyclooxygenase-2 (COX-2) expression by mitochondrial-derived damage associated molecular patterns (mtDAMPs) results in the generation of prostaglandin E_2_ (PGE_2_) by neutrophils in response to exogenous treatment with arachidonic acid (AA). **(A)** Overview of experimental design of studies that investigated whether the mtDAMP-induced up-regulation of COX-2 resulted in PGE_2_ synthesis. **(B)** Following a 3 hour treatment with 40 µg/ml mtDAMPs or vehicle control, neutrophils were stimulated for 20 minutes with 20 µM AA, after which PGE_2_ concentrations in cell free supernatants were measured (n=10). **(C)** Inhibition of COX-2 activity in 40 µg/ml mtDAMP stimulated neutrophils by a 1 hour treatment with 10 µM NS398 significantly reduced their production of PGE_2_ when cultured with 20 µM AA (n=7). **(D)** Overview of experimental design of studies that investigated whether the PGE_2_ containing supernatants of AA and mtDAMP co-treated neutrophils could inhibit the production of tumour necrosis factor-alpha (TNF-α) by lipopolysaccharide (LPS) stimulated THP-1 cells. **(E)** TNF-α concentrations in supernatants of 1 µg/ml LPS challenged THP-1 monocytes pre-treated for 30 minutes with conditioned media obtained from 20 µM AA-treated neutrophils that had been pre-stimulated with 40 µg/ml mtDAMPs or vehicle (n=8). **(F)** TNF-α mRNA expression (relative to untreated controls) in 1 µg/ml LPS challenged THP-1 monocytes cultured in conditioned media obtained from 20 µM AA-treated neutrophils that had been pre-stimulated with 40 µg/ml mtDAMPs or vehicle, or 40 µg/ml mtDAMPs in the presence or absence of 10 µM of the selective COX-2 inhibitor NS398 (n=6). **(G)** TNF-α concentrations in supernatants of 1 µg/ml LPS challenged THP-1 monocytes pre-treated with conditioned media obtained from 20 µM AA-treated neutrophils that had been pre-stimulated with 40 µg/ml mtDAMPs in the presence or absence of 10 µM of the selective COX-2 inhibitor NS398 (n=6). *p<0.05, **p<0.01, ***p<0.001.

To determine whether the mtDAMP-induced up-regulation of COX-2 expression in neutrophils resulted in the synthesis of PGE_2_, we incubated mtDAMP treated neutrophils with exogenous AA and measured PGE_2_ levels in cell free supernatants (**Figure 11A**). Compared to vehicle controls, mtDAMP pre-treated neutrophils generated significantly higher levels of PGE_2_ when cultured with AA (p<0.01; **Figure 11B**). The dependency on COX-2 for this PGE_2_ production was demonstrated by the significantly reduced concentrations of PGE_2_ detected in supernatants of mtDAMP stimulated neutrophils that had been pre-treated with the selective COX-2 inhibitor NS398 prior to culture with AA (p<0.05; **Figure 11C**).

To investigate whether the PGE_2_ containing supernatants of AA and mtDAMP co-treated neutrophils could modulate the anti-microbial functions of monocytes, we incubated THP-1 cells in these supernatants prior to LPS challenge (**Figure 11D**). Compared to vehicle-treated controls, supernatant-treated THP-1 cells exhibited significantly reduced TNF-α secretion (p<0.001; **Figure 11E**) and gene transcription (p<0.05; **Figure 11F**) in response to LPS stimulation. This impairment was dependent upon PGE_2_ as no reduction in TNF-α gene transcription or secretion was detected for LPS challenged THP-1 cells cultured in supernatants derived from neutrophils treated with mtDAMPs and the COX-2 specific inhibitor NS398 (p<0.05; **Figures 11F, G**).

### Inhibition of COX-2 and/or PKA signalling enhances *ex vivo* innate immune function post-trauma

**Figure 12:**
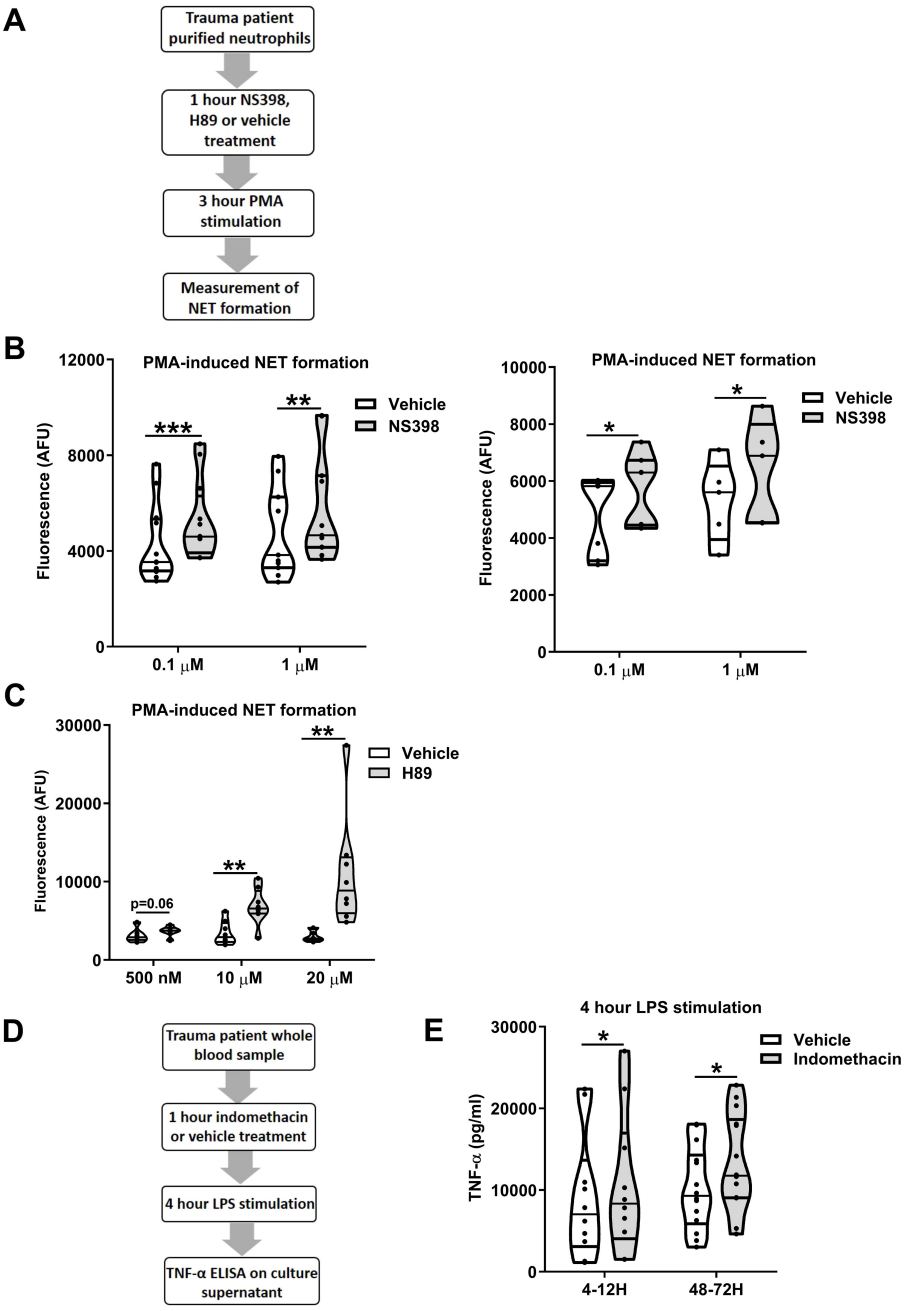
*Ex vivo* inhibition of cyclooxygenases (COX) or protein kinase A (PKA) enhances the anti-microbial activity of leukocytes post-trauma. **(A)** Overview of study design for neutrophil-based experiments investigating the role of COX-2 and protein kinase A signalling in neutrophil dysfunction post-trauma. **(B)** Pre-treatment of neutrophils isolated from trauma patients 4-12 (left panel) and 48-72 (right panel) hours post-injury with the selective COX-2 specific inhibitor NS398 (0.1-1 µM) significantly enhanced extracellular trap formation by neutrophils stimulated for 3 hours with 25 nM phorbol 12-myristate 13-acetate (PMA). For 4-12 hour samples, n = 12 and n = 11 for 0.1 µM and 1 µM NS398 doses respectively. For 48-72 hour samples, n = 7 and n = 5 for 0.1 µM and 1 µM NS398 doses respectively. **(C)** Pre-treatment of neutrophils isolated from trauma patients 4-12 hours post-injury with the PKA inhibitor H89 (0.5-20 µM) significantly enhanced extracellular trap formation by neutrophils stimulated for 3 hours with 25 nM PMA. Data were generated from blood samples collected from 6 (0.5 µM) and 8 (10, 20 µM) trauma patients. **(D)** Overview of study design for experiments investigating the role of COX signalling in trauma-induced endotoxin tolerance. **(E)** Tumour necrosis factor-alpha (TNF-α) concentrations in cell free supernatants of lipopolysaccharide (LPS) challenged (100 ng/ml, 4 hours) whole blood samples pre-treated for 1 hour with 10 µM of the COX inhibitor indomethacin or vehicle control (right panel). Samples were obtained from trauma patients 4-12 hours (n=10) and 48-72 hours (n=14) post-injury. *p<0.05, **p<0.01, ***p<0.001. AFU, arbitrary fluorescence units.

To examine whether modulation of the PGE_2_ pathway could restore *ex vivo* innate immune function post-injury, we treated neutrophils isolated from trauma patients 4-12 and 48-72 hours post-injury with NS398 prior to PMA challenge (**Figure 12A**). At both sampling time-points, NS398 pre-treatment, when compared to vehicle control, significantly enhanced PMA-induced NET production (p<0.05; **Figure 12B**). A significant increase in NET formation in response to PMA stimulation was also observed for trauma patient neutrophils that were pre-treated with the PKA inhibitor H89 (p<0.01; **Figure 12C**).

In whole blood assays, pre-treatment of samples obtained from trauma patients 4-12 and 48-72 hours post-injury with the non-selective COX inhibitor indomethacin resulted in significantly increased TNF-α production following LPS stimulation (p<0.05; **Figures 12D, E**).

## Discussion

By counteracting the hyper-inflammatory SIRS response triggered by major traumatic injury, the CARS response aims to restore immunological homeostasis (1). However, when dysregulated, an exaggerated CARS response of increased duration and/or magnitude culminates in a state of systemic immunosuppression that predisposes patients to the development of HAIs (5). Although of clinical relevance, the mechanisms that underpin this state of hyper resolution are poorly understood. Here, in a prospective study of 95 trauma patients, we examined the impact of injury on the biology of PGE_2_, a recently assigned SAMP that has been proposed as a potential mediator of systemic immunosuppression in critically-ill patients (34).

Our measurement of circulating PGE_2_ concentrations during the ultra-early and acute post-injury phases revealed the levels of this eicosanoid peaked in the pre-hospital setting. This observation aligns with findings of a previous study of polytrauma patients where the highest post-injury concentrations of PGE_2_ were also recorded in blood samples acquired at the scene of injury (51). Elevated levels of PGE_2_ have also been observed in thermally-injured patients (22, 23) and in subjects with acute TBI (21), with those presenting with higher levels experiencing poorer clinical recovery (21). Albumin, the most abundant protein in plasma, binds to and inactivates PGE_2_ (42, 43). At all our sampling time-points, trauma patients presented with lower concentrations of albumin when compared to HC. A consequence of haemorrhage, hypermetabolism, inflammation and/or capillary leak (52, 53), we suggest that injury-induced hypoalbuminemia would increase the bioavailability of PGE_2_, thereby promoting systemic immunosuppression. Interestingly, albumin supplementation in patients with advanced cirrhosis, who present with innate immune dysfunction and experience frequent infections, has been shown to restore immune function *ex vivo*, whilst murine models of bacterial peritonitis have demonstrated that administration of albumin can lower plasma PGE_2_ levels and significantly enhance bacterial clearance rates (54). Based on these data, albumin has been proposed to be an immune restorative drug, and albumin supplementation touted as a potential therapeutic strategy by which to restore immune competency and prevent nosocomial infections in patients with end stage liver disease (54). Whether such an approach would be beneficial in counteracting injury-induced immunosuppression requires investigation. For example, whilst safe and tolerated in patients with extracranial injuries (ECI), the use of albumin solution as a resuscitation fluid is not recommended in patients with TBI (52, 55, 56). Thus, if to be pursued in future studies, ECI patients with hypoalbuminemia would appear to be the most suitable cohort in which to test albumin replenishment as a means of restoring immune function post-trauma.

Suggesting that injured tissues may be a potential source of circulating PGE_2_ post-injury, we detected significantly elevated concentrations of the cell damage markers LDH and HMGB-1 in blood samples obtained at the scene of injury, the time-point at which peak PGE_2_ levels were observed. In support of this hypothesis, release of PGE_2_ has been shown to be a feature common to various forms of cell death such as necrosis, pyroptosis and apoptosis (57). Besides damaged tissues, activated leukocytes are also likely to have contributed to the post-trauma increase in circulating PGE_2_, with previous studies reporting enhanced *ex vivo* synthesis of this eicosanoid by macrophages, neutrophils, monocytes and PBMCs isolated from severely-injured patients or mice (25–28, 58–61). Our interrogation of trauma-induced changes in the expression of enzymes involved in PGE_2_ synthesis revealed a post-injury increase in the mRNA levels of cPLA_2_ in PBMCs and in COX-2 in both PBMCs and neutrophils. These observations confirm findings reported by previous studies in the settings of burns and trauma, where post-injury elevations in COX-2 protein expression were also described (24, 25, 31, 58–60). As the promoter regions of the genes encoding COX-2 and cPLA_2_ contain binding sites for NFκβ, the increased DNA binding activity of this transcription factor, which has been reported to occur in leukocytes post-injury (59) could explain the trauma-induced up-regulation in COX-2 and cPLA_2_ mRNA levels we describe here.

Our *ex vivo* assessments of innate immune function revealed an immediate and persistent state of immune suppression post-trauma, with endotoxin tolerant monocytes detected in blood samples acquired at our pre and in-hospital sampling time-points. Correlative analyses revealed no relationship between a patients ISS and cytokine production by LPS challenged monocytes post-injury. However, when compared to HC, endotoxin tolerant monocytes were detected in both moderate (ISS <16) and severely (ISS>16)-injured patients. Interestingly, by 48-72 hours post-injury, we observed no impairment in TNF-α production by monocytes isolated from moderately-injured patients subjected to an 18 hour *ex vivo* LPS challenge, which suggests an earlier recovery of immune function in patients with milder injuries. These observations are in line with those of a previous study of 58 trauma patients that reported: (i) significantly impaired IL-1β production by LPS treated monocytes isolated from trauma patients with ISS of <16 and >16 when compared to HC, (ii) no significant differences in IL-1β generation between trauma patients based on ISS and (iii) an earlier recovery of LPS-induced cytokine production by monocytes isolated from patients that had sustained minor injuries (62). The impairment we detected in monocyte function was accompanied by a significant post-injury reduction in ROS generation and phagocytosis by *E.coli* challenged neutrophils. *E.coli* is the predominant cause of UTIs in trauma patients, and one of the most common pathogens recovered from wound and respiratory isolates of injured patients that develop nosocomial infections (63–65). Thus, neutrophil dysfunction post-trauma could increase the susceptibility of patients to *E.coli* infections. Future studies should extend upon these observations to examine whether trauma also negatively impacts upon the effector functions elicited by neutrophils towards Gram positive bacteria (e.g. Staphylococcus aureus). Implicated in the development of common HAIs such as pneumonia, as well as severe HAIs (e.g. osteomyelitis), understanding the importance of neutrophil functions in the host response to gram positive bacteria is of clinical relevance given that this type of bacteria represents a significant cause of morbidity amongst hospitalised trauma patients (66, 67).

In this study, we were able to replicate features of the CARS response in leukocytes isolated from HC by exposing them to concentrations of PGE_2_ that we and others have measured in the circulation of severely-injured patients (21, 22). Whilst we are not the first study to report upon PGE_2_-induced suppression of monocyte and neutrophil anti-microbial functions, previous studies have used PGE_2_ at concentrations that exceed the physiological levels produced following major trauma (16–19, 33). Thus, our results suggest that the generation of PGE_2_ post-injury may contribute to the onset of systemic immune suppression. However, raised PGE_2_ levels will not be the only explanation for the development of neutrophil and monocyte dysfunction post-trauma, with other factors, such as the presence in circulation of immune tolerising mtDAMPs and the release of immature neutrophils from the bone marrow also likely to play a role (68, 69). Another feature of the CARS response is reduced expression of the antigen presenting molecule HLA-DR by monocytes, a phenotypic alteration that has been linked to an increased susceptibility of critically ill patients to HAIs (70–72). As exposure to 1 ng/ml PGE_2_ has been shown to reduce monocyte HLA-DR expression *in vitro* (73), this component of the CARS response could also be attributable in part to the post-injury elevation in PGE_2_ levels. Alongside its direct effects, it is conceivable that PGE_2_ may also promote post-trauma immune suppression indirectly by enhancing the inhibitory/suppressive effects of other leukocytes. For example, studies have shown exposure to PGE_2_ can promote the differentiation of resting T cells into FOXP3^+^ regulatory T cells (74) and potentiate the immunomodulatory functions of myeloid derived suppressor cells, which include the inhibition of natural killer cell cytotoxicity, suppression of T cell activation and induction of M2 macrophages (75–77).

Traumatic injury is associated with reactivation of dormant herpes viruses, which increases the susceptibility of hospitalised patients to the development of secondary bacterial infections (44–46). Studies have reported that levels of the anti-inflammatory cytokine IL-10 are elevated in trauma patients who experience viral reactivation (44), whilst IL-15, produced by myeloid cells, promotes anti-viral defence by stimulating natural killer cell effector functions and T cell cytotoxicity (47, 78). Here, we showed that prior exposure to PGE_2_ significantly increased IL-10 generation by MDMs following LPS challenge, and that this was accompanied by a significant impairment in the production of TNF-α. We found no effect of PGE_2_ exposure on either the expression by MDMs of CD215, a surface molecule that trans-presents IL-15 to NK cells, or on LPS-induced IL-15 gene transcription by primary human monocytes. Our observations of impaired TNF-α production and enhanced IL-10 generation by LPS challenged MDMs pre-treated with PGE_2_ are in accordance with results of previous studies (79, 80), and suggest that elevated concentrations of PGE_2_ at sites of tissue injury may create an immune suppressive environment that could potentially increase the risk of the reactivation of dormant herpes viruses by negatively impacting upon localised immune responses.

Promoting the resolution of inflammatory responses, the uptake of apoptotic neutrophils by macrophages is a key anti-inflammatory mechanism (48). Here, we found that prior treatment with PGE_2_ had no impact on either the ability of MDMs to perform efferocytosis or upon their expression of SIRP-α, a negative regulator of efferocytosis (49). As our *ex vivo* analysis of blood samples acquired from trauma patients within minutes of injury revealed evidence of impaired immune function and significantly elevated concentrations of PGE_2_, we chose to study the efferocytic capacity and surface phenotype of MDMs subjected to a 60 minute *in vitro* pre-treatment with PGE_2_. This short-term exposure, coupled with our use of MDMs cultured in conditions that would induce a firm M1 bias, could therefore explain the lack of an effect we detected for PGE_2_ exposure on these two parameters. Future studies that investigate the effects that extended PGE_2_ treatment times have on efferocytosis and MDM phenotype would help address this theory. Moreover, whilst rodent based studies have examined how the uptake of dying neurons by microglia and peripheral derived macrophages regulates neuroinflammation following traumatic brain injury (81), no study to our knowledge has addressed whether the uptake of apoptotic neutrophils by macrophages is altered in human subjects post-injury. Such studies are worthy of further investigation given that any impairments in this process could, by promoting dysregulation in the resolution of inflammatory responses, be a contributory factor for the state of persistent inflammation that develops in critically-ill patients that experience complicated clinical outcomes (82).

PGE_2_ mediates its immune suppressive effects by binding to the surface expressed receptors EP2 and EP4. Our experiments with the selective EP2 and EP4 receptor antagonists AH6809 and L161,982 respectively showed that signalling through EP4 was responsible for the PGE_2_-induced suppression of TNF-α production by LPS challenged whole blood leukocytes. This observation is in line with previous studies that also found signalling through the EP4 receptor promoted the PGE_2_-induced inhibition of TNF-α production by LPS challenged monocytes and macrophages (79, 83–85). It has been suggested that the EP4 receptor is critical in mediating the suppression of LPS-induced TNF-α generation by low doses of PGE_2_, whereas, due to its lower affinity, signalling through the EP2 receptor only becomes relevant when PGE_2_ is present at higher levels (85). The use of endogenous physiological concentrations of PGE_2_ in our experimental assays could therefore potentially explain why low doses of PGE_2_ were able to inhibit LPS-induced TNF-α production by monocytes pre-treated with EP2, but not EP4 antagonists. Suggesting that leukocytes may be more sensitive to the immune inhibitory effects of PGE_2_ post-trauma, we found EP4 mRNA levels were significantly higher in PBMCs isolated from trauma patients in the pre-hospital setting. Our observation of increased EP4 expression post-injury is in contrast to the previous work of Strong et al. who detected significantly lower EP4 mRNA levels in LPS stimulated PBMCs isolated from 10 trauma and burns patients within 12 hours of injury (25). This variation in sample timing (pre-hospital Vs post-hospital), patient cohort (trauma and burns Vs trauma only) and experimental design (resting Vs LPS challenged PBMCs) could explain these contrasting findings. Coupled to the G_αs_ protein, activation of EP4 increases intracellular levels of the second messenger cAMP via activation of adenylate cyclase. Demonstrating a role for this pathway in PGE_2_-induced suppression of cytokine production, we found the PDE4 inhibitor rolipram potentiated the suppressive effect of exogenous PGE_2_ treatment on TNF-α production by LPS stimulated monocytes. Moreover, direct activation of adenylate cyclase by treatment of whole blood with forskolin significantly reduced TNF-α synthesis triggered by exposure to LPS. Alongside monocytes, PGE_2_ treatment significantly impaired NET formation and ROS production by neutrophils stimulated with PMA, defects that have previously been reported for neutrophils isolated from major trauma patients (3, 4). Whilst not investigated here, current literature points towards both EP2 and EP4 signalling in mediating the inhibition of neutrophil anti-microbial activity by PGE_2_. For example, prior exposure to EP2 and EP4 receptor antagonists has been shown to reverse PGE_2_-mediated impairments in neutrophil NET generation (16, 17), whilst treatment of neutrophils from healthy volunteers with EP2 and EP4 receptor agonists can recapitulate the post-trauma reduction in NET formation (17).

*In vivo* and *ex vivo* studies have shown that targeting PGE_2_ production can restore immune function post-injury (30, 32, 58). Of particular significance are data that found that treatment with either the selective COX-2 inhibitor NS398 or the broad acting COX inhibitor indomethacin improved survival rates in mice subjected to trauma and infectious challenge (31, 32), and lowered the rates of early opportunistic infections in patients undergoing major surgery (86). In addition to demonstrating the restoration of leukocyte cytokine production post-injury by *ex vivo* treatment of whole blood with indomethacin, we showed for the first time that inhibition of COX-2 could increase neutrophil anti-microbial activity post-trauma. Specifically, we found that pre-treatment of neutrophils with NS398 significantly enhanced PMA-induced NET production. Increased NET formation was also observed for PMA stimulated neutrophils pre-treated with the PKA antagonist H89. This restoration of NET generation via manipulation of the PGE_2_ signalling pathway is in agreement with a previous study that enrolled hematopoietic stem cell transplant patients and found *ex vivo* treatment with H89 augmented NET production triggered by PMA stimulation (16). Data are therefore accumulating that suggests a potential role for COX inhibitors in reversing immune dysfunction (58). However, more work is needed to establish whether the benefits of this therapeutic approach would outweigh the negative clinical outcomes linked to the use of COX-2 inhibitors such as delayed wound healing (87).

It was recently suggested that DAMP activated leukocytes are a source of PGE_2_ post-trauma (34). To test this hypothesis, we exposed leukocytes from healthy volunteers to mtDAMPs, a collection of DNA, proteins and lipids whose circulating concentrations are elevated in the minutes, hours and days following injury (2, 4, 88). Compared to vehicle controls, we found significantly increased COX-2 mRNA and protein expression in neutrophils and monocytes treated with 40 µg/ml mtDAMPs, which in the case of neutrophils, resulted in enhanced PGE_2_ production in the presence of exogenous AA. Importantly, in crossover experiments, supernatants derived from mtDAMP and AA co-treated neutrophils suppressed TNF-α production and gene transcription by LPS stimulated monocytes. Via the use of pharmacological inhibitors, we demonstrated that N-formylated peptides within mtDAMP preparations were responsible for the induction of COX-2 expression, which was dependent upon ERK1/2, P38 and Pi3-kinase signalling. This formyl peptide, MAPK and Pi3-kinase driven increase in COX-2 expression mirrors results of previous studies that reported the same signalling pathways were involved in inducing COX-2 protein expression in neutrophils treated with bacterial-derived N-formyl peptides, which signal through the same surface receptor as mtDAMPs (35–37, 89). An immediate and persistent exposure to mtDAMPs could therefore be a contributory factor underlying the post-trauma increase in COX-2 mRNA and protein levels that we and others have observed in neutrophils and monocytes (24, 25).

Our study has limitations. Conducted at a single major trauma centre, our *ex vivo* analyses were performed on a relatively small number of patient samples, meaning that our findings require validation in larger independent cohorts. Our use of PMA in NET and ROS assays may be deemed inappropriate given that it is a pharmacological agent of non-physiological relevance. However, PMA is a potent stimulus that has been used in previous studies that have investigated the impact of PGE_2_ and traumatic injury on NET generation (3, 4, 16, 17, 90). Using this stimulus therefore enabled us to compare our observations to those of previous publications. Similarly, the circulating concentrations of mtDAMPs post-trauma are currently unknown. Our choice of 40 µg/ml was based on previous *in vitro* studies that had used this dose in experiments that examined how exposure to mtDAMPs modulates innate immune function (4, 38, 39, 91). Finally, our experiments focussed solely upon the modulation of innate immune responses by PGE_2_. Given that *in vitro* exposure to this eicosanoid has been shown to suppress proliferation and cytokine production by T cells isolated from HC (92–94), features that are reminiscent of the immune profile of traumatically-injured patients (95), it would be of interest for future studies to investigate whether the post-injury generation of PGE_2_ by neutrophils, monocytes and/or macrophages may contribute to the suppression of adaptive immune responses following major trauma.

In conclusion, our study has provided evidence that suggests PGE_2_ is a potential mediator of the immediate state of systemic innate immune dysfunction that develops following major trauma. Moreover, our finding that exposure to mtDAMPs can promote both COX-2 expression and PGE_2_ production by leukocytes provides a potential mechanistic explanation for the emerging concept of mtDAMP-induced immune tolerance (68). Given that *ex vivo* treatment of leukocytes with inhibitors targeting either COX-2 or adenylate cyclase enhanced innate immune responses post-injury, it would be of interest for future studies to examine whether manipulation of PGE_2_ production and/or signalling could prevent the development of HAI amongst critically-ill patients by restoring innate immune function. These studies could be performed alongside others that investigate whether albumin supplementation in specific cohorts of trauma patients such as those with ECI could, by decreasing PGE_2_ bioavailability, reverse trauma-induced suppression of monocyte and/or neutrophil anti-microbial activity.

## Data Availability

The raw data supporting the conclusions of this article will be made available by the authors, without undue reservation.

## Supplementary material

Supplementary Figure 1 Determination of purity of isolated neutrophil and monocyte preparations. The purity of neutrophils and monocytes isolated from whole blood by Percoll density gradient centrifugation and magnetic assisted cell sorting technology respectively were determined using a Sysmex XN-1000 automated differential haematology analyser. **(A-C)** Representative plots generated from the Sysmex XN-1000 that show the leukocyte profiles of whole blood **(A)**, purified neutrophils **(B)** and purified monocytes **(C)**. In **(A)**, labelled cell populations are as follows: 1, Neutrophils; 2, Monocytes; 3, Lymphocytes. The accuracy of the XN-1000 for estimates of cell purity were confirmed by Giemsa staining of isolated neutrophils **(D)** and monocytes **(E)**.

Supplementary Figure 2 Flow cytometry gating strategy to study neutrophil oxidative burst and phagocytic activity. **(A-D)** Gating strategy for data acquisition and analysis of neutrophil oxidative burst in whole blood samples. **(A)** Using a propidium iodide (PI) based DNA staining solution that generates a positive signal for human diploid cells in the PE channel, a “live” gate (M1) is set on total leukocytes within a whole blood sample. **(B)** Based on their distinct forward scatter (FSC)/sideward scatter (SSC) properties, a gate is set on the neutrophil population (E1). **(C)** A forward scatter (FSC)/width dot plot is created to select for “single cells” (gate R3) and to exclude neutrophil aggregates. 10,000 “single” neutrophils are collected within the R3 gate for each experimental condition. **(D)** The mean fluorescence signal generated from the oxidation of dihydrorhodamine 123 by reactive oxygen species produced by untreated (black histogram), *E.coli* (blue histogram) or phorbol 12-myristate 13-acetate (PMA; red histogram) stimulated neutrophils is recorded in the FITC channel. **(E-H)** Gating strategy for data acquisition and analysis of neutrophil phagocytic activity in whole blood samples. **(E)** PI based DNA stain to distinguish leukocytes (gate M1) from bacteria. **(F)** Neutrophils (gate P2) are identified based on their FSC/SSC properties. **(G)** 10,000 “single” neutrophils are gated to exclude “doublet” cells (gate R3). **(H)** The phagocytic activity of neutrophils incubated with FITC-labelled opsonised *E.coli* at 0°C (black histogram) and 37°C (red histogram) is recorded as the percentage of FITC positive cells (gate M2) and mean fluorescence intensity of the FITC channel.

Supplementary Figure 3 Flow cytometry gating strategy for efferocytosis assay. **(A)** Apoptotic neutrophils stained with CellTracker™ Deep Red were ran in all experimental assays to allow for cell gating on forward scatter(FS)/sideward scatter(SS) plots (left panel). Using this gate, the threshold for positive fluorescence was established on APC (FL7) histograms (right panel). **(B)** Samples comprised of monocyte-derived macrophages (MDMs) only were ran in each experimental assay to allow for gating of MDM populations on FS/SS plots (left panel) and to ensure that MDMs alone did not fluoresce above the positive threshold set on the APC channel based on the gating of samples of neutrophils alone (right panel). **(C)** In test samples, which comprised of MDMs co-cultured with apoptotic neutrophils, MDMs were gated (left panel) and the percentage exhibiting positive APC fluorescence, which is used as the readout for neutrophil uptake (efferocytosis) recorded (right panel). The percentage of APC positive MDMs recorded in co-culture samples where MDMs had been pre-treated with the actin polymerisation inhibitor cytochalasin D was subtracted from the percentage of APC positive MDMs recorded in the test sample to generate an efferocytosis index.

Supplementary Figure 4 Serum prostaglandin E_2_ (PGE_2_) and albumin concentrations in male and female trauma patients. **(A, B)** At our three post-injury sampling time-points (pre-hospital, 4-12 hours and 48-72 hours post-injury) serum concentrations of PGE_2_ **(A)** and albumin **(B)** were recorded and their levels compared between male and female patients. Number of samples analysed are as follows: PGE_2_; pre-hospital, males=44, females=8; 4-12 hours, males=42, females=7; 48-72 hours, males=41, females=6. Albumin; pre-hospital, males=30, females=4; 4-12 hours, males=29, females=4; 48-72 hours, males=26, females=4.

Supplementary Figure 5 Tumour necrosis factor-alpha (TNF-α) production by lipopolysaccharide (LPS) challenged whole blood leukocytes. Intracellular TNF-α levels in LPS challenged (100 ng/ml, 4 hours) whole blood leukocytes measured as percentage TNF-α positive cells (left panel) or median fluorescence intensity (MedFI) readings (right panel). Cell types were identified based on surface phenotype; CD15^+^, neutrophils;CD3^+^, T lymphocytes; CD19^+^, B lymphocytes and CD14^+^, monocytes. Data were generated from 5 independent experiments. ***p<0.0005.

Supplementary Table 1 Primer sequences used for real-time polymerase chain reaction experiments. COX-2, Cyclooxygenase-2; cPLA_2_, Cytosolic phospholipase A2; EP, E-prostanoid; MPGES-1, Microsomal prostaglandin E synthase-1; TNF-α, Tumour necrosis factor-alpha.

Supplementary Table 2 Comparison of tumour necrosis factor-alpha (TNF-α) concentrations measured in supernatants of whole blood leukocytes isolated from healthy controls (HC) and trauma patients (<1, 4-12 and 48-72 hours post-injury) with an injury severity score <16 or ≥16 following a 4 or 18 hour stimulation with lipopolysaccharide (LPS). Data are presented as mean ± standard error. **p<0.005, ***p<0.0005 Vs. HC. HC, Healthy control, ISS, Injury severity score; LPS, Lipopolysaccharide, TNF-α, Tumour necrosis factor-alpha.

Supplementary Table 3 Paired measurements of prostaglandin E_2_ (PGE_2_) concentrations and oxidative burst response of *E.coli* or phorbol 12-myristate 13-acetate (PMA) stimulated neutrophils measured for trauma patients 48-72 hours post-injury. MFI, Mean fluorescence intensity; PGE_2_, Prostaglandin E_2_; PMA, phorbol 12-myristate 13-acetate.

Supplementary Table 4 Comparison of the expression of enzymes and receptors involved in prostaglandin E2 synthesis and signalling in peripheral blood mononuclear cells (PBMCs) and neutrophils isolated from male and female trauma patients. COX-2, Cyclooxygenase-2; cPLA_2_, cytosolic phospholipase A2; EP2, E prostanoid receptor 2; EP4, E prostanoid receptor 4; mPGES-1, microsomal prostaglandin E synthase-1; PBMCs, Peripheral blood mononuclear cells. Data are presented as expression relative to values recorded for healthy controls. Significant differences in gene expression between males and females are indicated in bold font.
