# Supplementary figures and images for: The impact of trauma relevant concentrations of prostaglandin E2 on the anti-microbial activity of the innate immune system
Source: Front Immunol. 2024 Oct 22;15:1401185. doi: 10.3389/fimmu.2024.1401185 (PMC11535544; doi:10.3389/fimmu.2024.1401185)

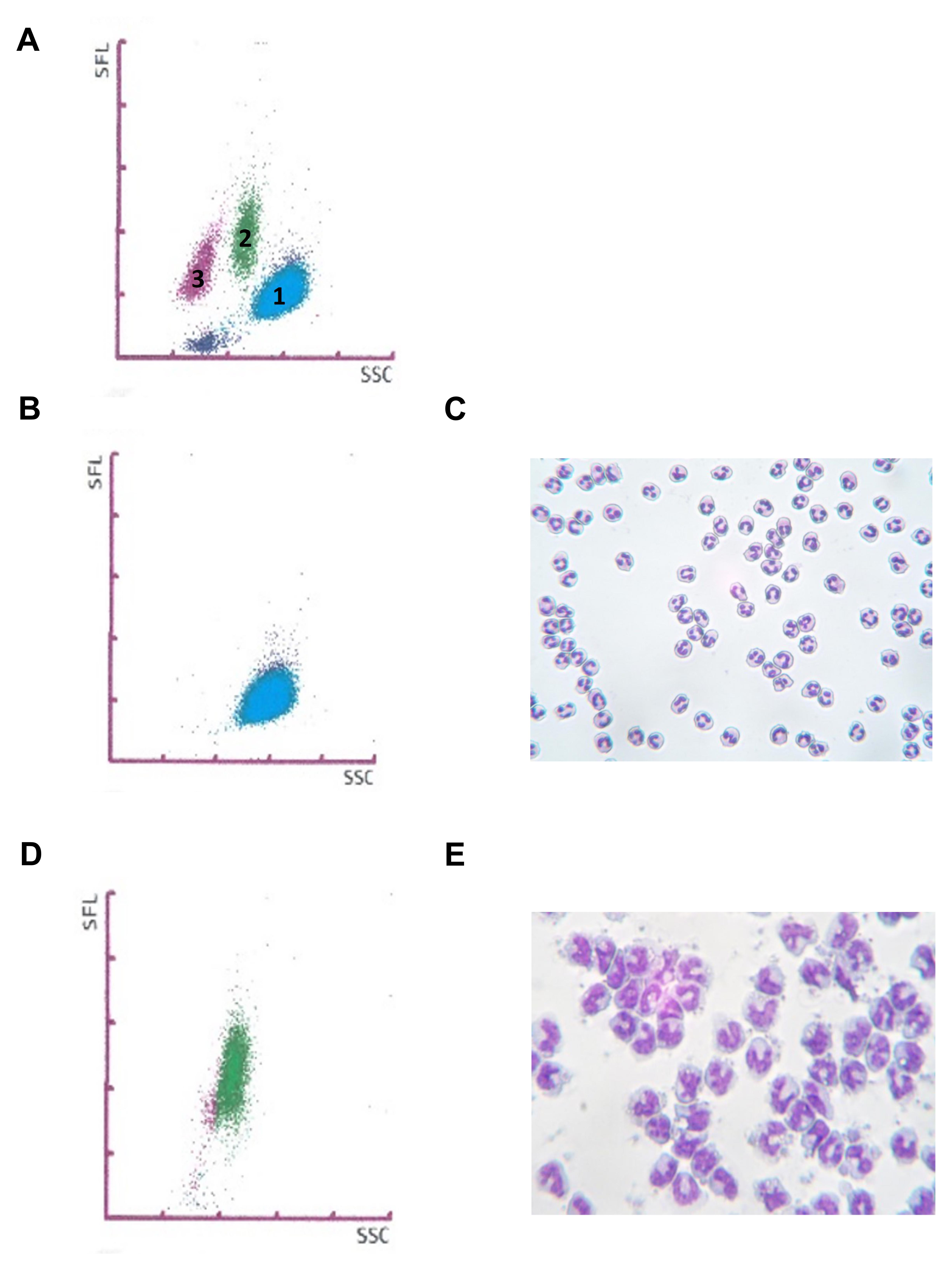

Supplement: Supplementary Figure 1 — Determination of purity of isolated neutrophil and monocyte preparations. The purity of neutrophils and monocytes isolated from whole blood by Percoll density gradient centrifugation and magnetic assisted cell sorting technology respectively were determined using a Sysmex XN-1000 automated differential haematology analyser. (A-C) Representative plots generated from the Sysmex XN-1000 that show the leukocyte profiles of whole blood (A), purified neutrophils (B) and purified monocytes (C). In (A), labelled cell populations are as follows: 1, Neutrophils; 2, Monocytes; 3, Lymphocytes. The accuracy of the XN-1000 for estimates of cell purity were confirmed by Giemsa staining of isolated neutrophils (D) and monocytes (E). [file Image1.jpeg]

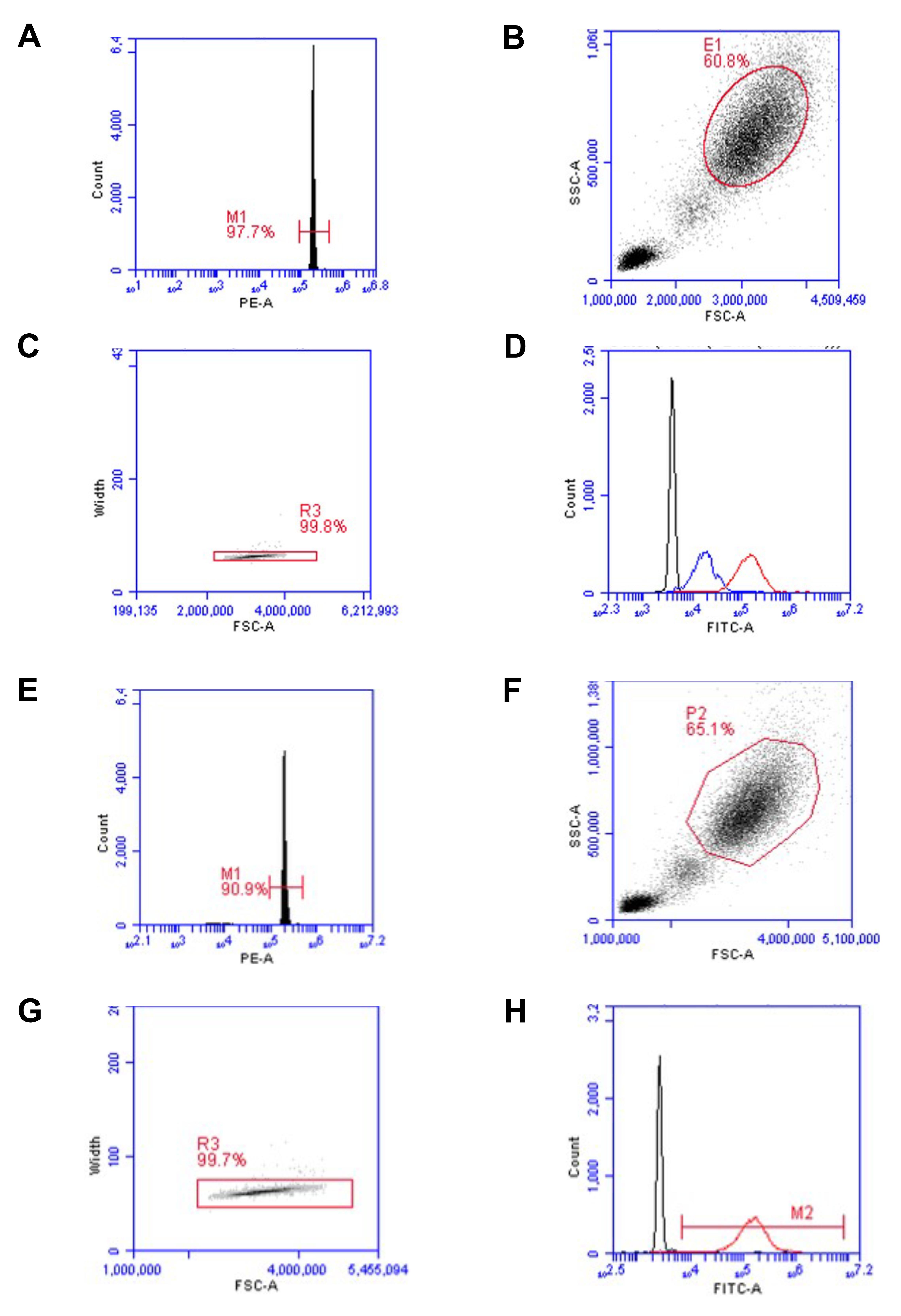

Supplement: Supplementary Figure 2 — Flow cytometry gating strategy to study neutrophil oxidative burst and phagocytic activity. (A-D) Gating strategy for data acquisition and analysis of neutrophil oxidative burst in whole blood samples. (A) Using a propidium iodide (PI) based DNA staining solution that generates a positive signal for human diploid cells in the PE channel, a “live” gate (M1) is set on total leukocytes within a whole blood sample. (B) Based on their distinct forward scatter (FSC)/sideward scatter (SSC) properties, a gate is set on the neutrophil population (E1). (C) A forward scatter (FSC)/width dot plot is created to select for “single cells” (gate R3) and to exclude neutrophil aggregates. 10,000 “single” neutrophils are collected within the R3 gate for each experimental condition. (D) The mean fluorescence signal generated from the oxidation of dihydrorhodamine 123 by reactive oxygen species produced by untreated (black histogram), E.coli (blue histogram) or phorbol 12-myristate 13-acetate (PMA; red histogram) stimulated neutrophils is recorded in the FITC channel. (E-H) Gating strategy for data acquisition and analysis of neutrophil phagocytic activity in whole blood samples. (E) PI based DNA stain to distinguish leukocytes (gate M1) from bacteria. (F) Neutrophils (gate P2) are identified based on their FSC/SSC properties. (G) 10,000 “single” neutrophils are gated to exclude “doublet” cells (gate R3). (H) The phagocytic activity of neutrophils incubated with FITC-labelled opsonised E.coli at 0°C (black histogram) and 37°C (red histogram) is recorded as the percentage of FITC positive cells (gate M2) and mean fluorescence intensity of the FITC channel. [file Image2.jpeg]

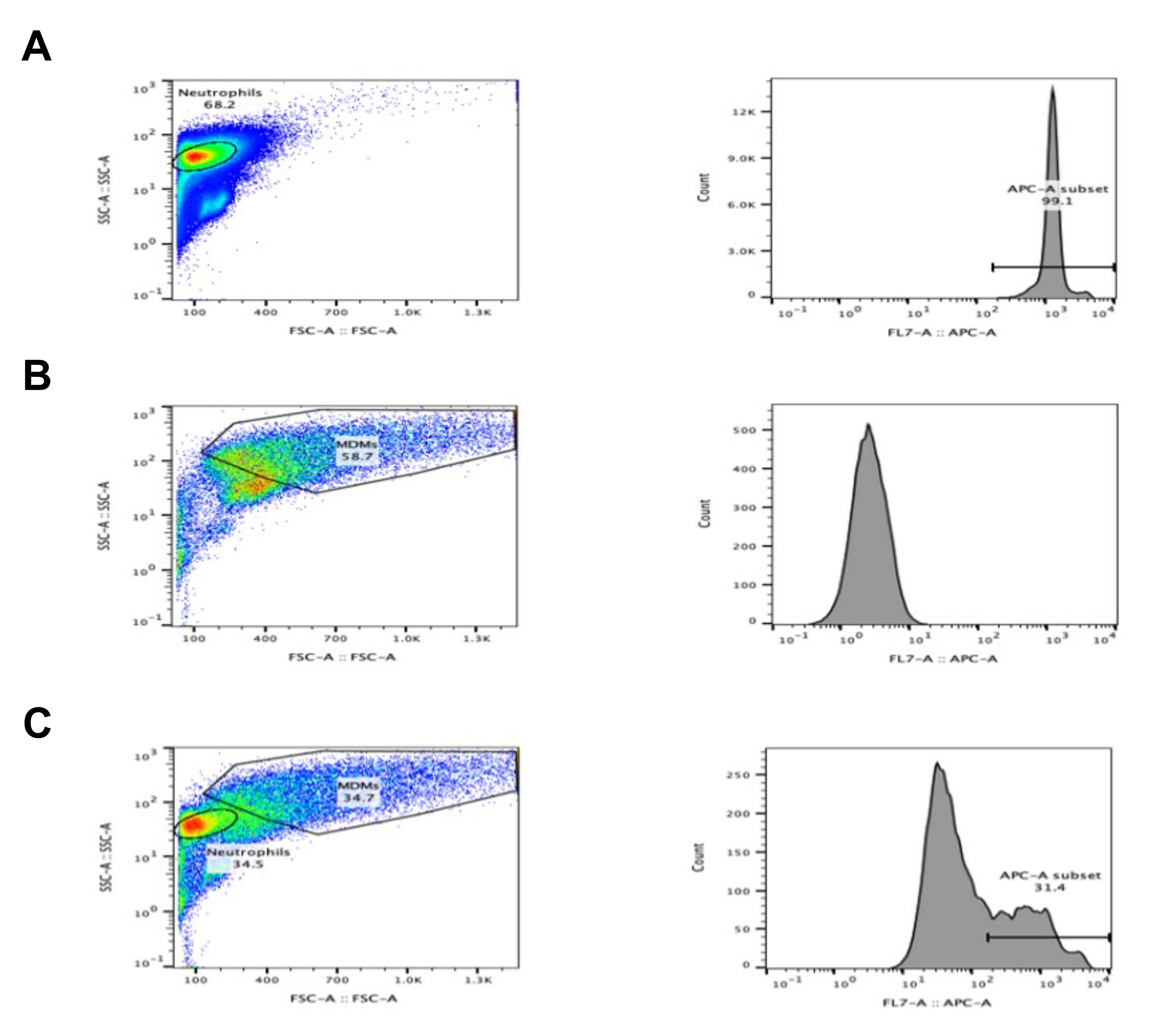

Supplement: Supplementary Figure 3 — Flow cytometry gating strategy for efferocytosis assay. (A) Apoptotic neutrophils stained with CellTracker™ Deep Red were ran in all experimental assays to allow for cell gating on forward scatter(FS)/sideward scatter(SS) plots (left panel). Using this gate, the threshold for positive fluorescence was established on APC (FL7) histograms (right panel). (B) Samples comprised of monocyte-derived macrophages (MDMs) only were ran in each experimental assay to allow for gating of MDM populations on FS/SS plots (left panel) and to ensure that MDMs alone did not fluoresce above the positive threshold set on the APC channel based on the gating of samples of neutrophils alone (right panel). (C) In test samples, which comprised of MDMs co-cultured with apoptotic neutrophils, MDMs were gated (left panel) and the percentage exhibiting positive APC fluorescence, which is used as the readout for neutrophil uptake (efferocytosis) recorded (right panel). The percentage of APC positive MDMs recorded in co-culture samples where MDMs had been pre-treated with the actin polymerisation inhibitor cytochalasin D was subtracted from the percentage of APC positive MDMs recorded in the test sample to generate an efferocytosis index. [file Image3.jpeg]

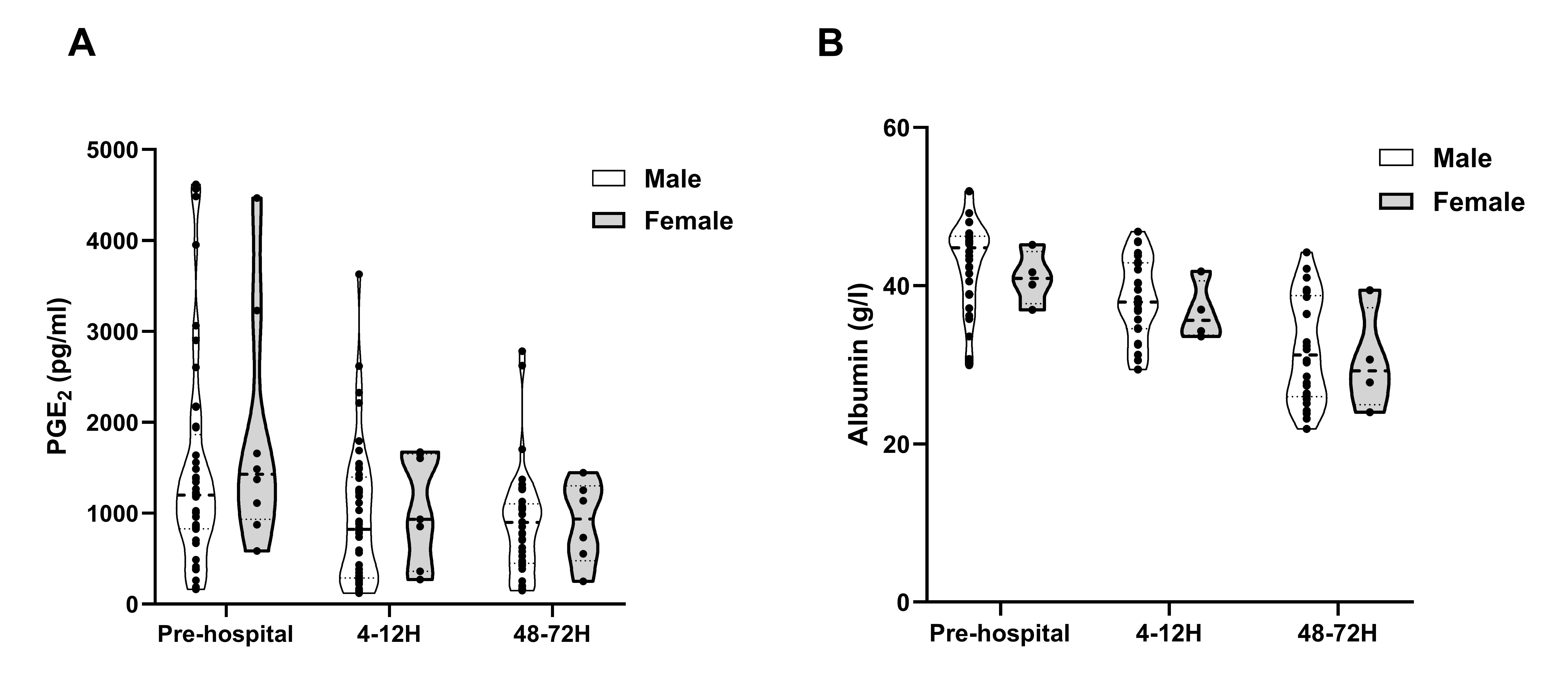

Supplement: Supplementary Figure 4 — Serum prostaglandin E2 (PGE2) and albumin concentrations in male and female trauma patients. (A, B) At our three post-injury sampling time-points (pre-hospital, 4-12 hours and 48-72 hours post-injury) serum concentrations of PGE2 (A) and albumin (B) were recorded and their levels compared between male and female patients. Number of samples analysed are as follows: PGE2; pre-hospital, males=44, females=8; 4-12 hours, males=42, females=7; 48-72 hours, males=41, females=6. Albumin; pre-hospital, males=30, females=4; 4-12 hours, males=29, females=4; 48-72 hours, males=26, females=4. [file Image4.jpeg]

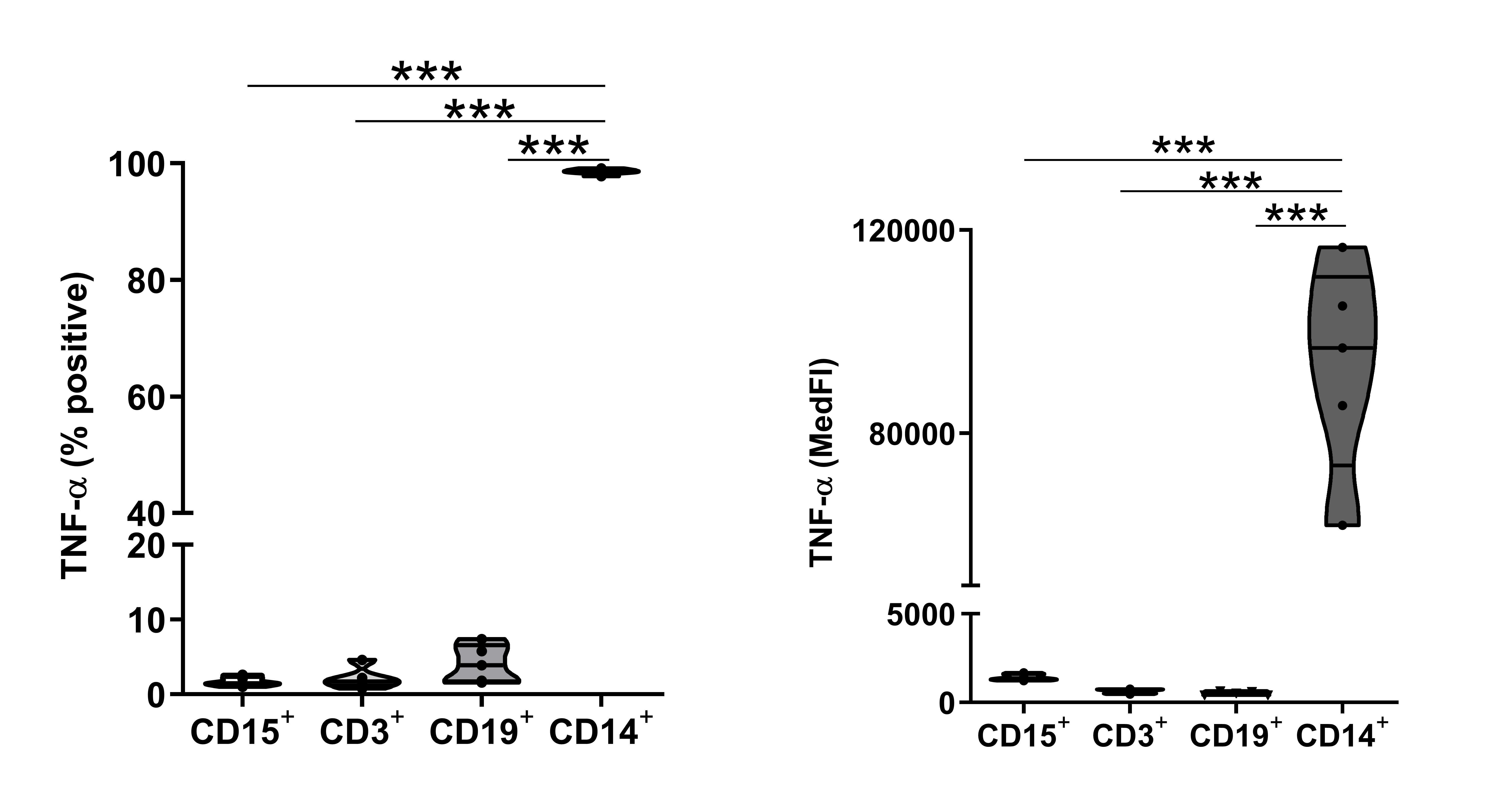

Supplement: Supplementary Figure 5 — Tumour necrosis factor-alpha (TNF-α) production by lipopolysaccharide (LPS) challenged whole blood leukocytes. Intracellular TNF-α levels in LPS challenged (100 ng/ml, 4 hours) whole blood leukocytes measured as percentage TNF-α positive cells (left panel) or median fluorescence intensity (MedFI) readings (right panel). Cell types were identified based on surface phenotype; CD15+, neutrophils;CD3+, T lymphocytes; CD19+, B lymphocytes and CD14+, monocytes. Data were generated from 5 independent experiments. ***p<0.0005. [file Image5.jpeg]
