# Supplementary material for: The impact of trauma relevant concentrations of prostaglandin E2 on the anti-microbial activity of the innate immune system
Source: Front Immunol. 2024 Oct 22;15:1401185. doi: 10.3389/fimmu.2024.1401185 (PMC11535544; doi:10.3389/fimmu.2024.1401185)
Supplement: Supplementary Table 1 — Primer sequences used for real-time polymerase chain reaction experiments. COX-2, Cyclooxygenase-2; cPLA2, Cytosolic phospholipase A2; EP, E-prostanoid; MPGES-1, Microsomal prostaglandin E synthase-1; TNF-α, Tumour necrosis factor-alpha. [file Table1.docx]

**Supplementary Table 1. Primer sequences used for real-time polymerase chain reaction experiments**

| **Target** | **Primer Sequence** | |
| --- | --- | --- |
|  | **Forward** | **Reverse** |
| **COX-2**  **cPLA_2_**  **MPGES-1**  **EP2**  **EP4**  **TNF-α**  **IL-15**  **18S** | 5’GTTTTGACATGGGTGGGAAC3’  5’GACGTGCTGGGAAGGTACAC3’  5’GGAACGACATGGAGACCATCTAC3’  5’GACCGCTTACCTGCAGCTGTAC3’  5’ACGCCGCCTACTCCTACATG3’  5’CCTCTCTCTAATCAGCCCTCTG3’  5’GGAAACCCCTTGCCATAGC3’  5’GTAACCCGTTGAACCCCATT3’ | 5’CCCTCAGACAGCAAAGCCTA3’  5’AGCCCACTGTCCACTACA3’  5’TCCAGGCGACAAAAGGGTTA3’  5’TGAAGTTGCAGGCGAGCA3’  5’AGAGGACGGTGGCGAGAAT3’  5’GAGGACCTGGGAGTAGATGAG3’  5’GATGGAAATACTTCTCAAATGTGGT3’  5’CCATCCAATCGGTAGTAGCG3’ |

COX-2, Cyclooxygenase-2; cPLA_2_, Cytosolic phospholipase A2; EP, E-prostanoid; MPGES-1, Microsomal prostaglandin E synthase-1; TNF-α, Tumour necrosis factor-alpha.
