# Supplementary material for: The impact of trauma relevant concentrations of prostaglandin E2 on the anti-microbial activity of the innate immune system
Source: Front Immunol. 2024 Oct 22;15:1401185. doi: 10.3389/fimmu.2024.1401185 (PMC11535544; doi:10.3389/fimmu.2024.1401185)
Supplement: Supplementary Table 2 — Comparison of tumour necrosis factor-alpha (TNF-α) concentrations measured in supernatants of whole blood leukocytes isolated from healthy controls (HC) and trauma patients (<1, 4-12 and 48-72 hours post-injury) with an injury severity score <16 or ≥16 following a 4 or 18 hour stimulation with lipopolysaccharide (LPS). Data are presented as mean ± standard error. **p<0.005, ***p<0.0005 Vs. HC. HC, Healthy control, ISS, Injury severity score; LPS, Lipopolysaccharide, TNF-α, Tumour necrosis factor-alpha. [file Table2.docx]

**Supplementary Table 2. Comparison of tumour necrosis factor-alpha (TNF-α) concentrations measured in supernatants of whole blood leukocytes isolated from healthy controls (HC) and trauma patients (<1, 4-12 and 48-72 hours post-injury) with an injury severity score <16 or ≥16 following a 4 or 18 hour stimulation with lipopolysaccharide (LPS).**

|  | **HC** | **ISS <16** | | | **ISS ≥16** | | |
| --- | --- | --- | --- | --- | --- | --- | --- |
|  |  | **<1H** | **4-12H** | **48-72H** | **<1H** | **4-12H** | **48-72H** |
| **4 Hour LPS stimulation**  *TNF-a (pg/ml)*  *n* | 29,871  **±**1,928  30 | 3,797 **±**1,166^***^  5 | 9,754 **±**3,445^**^  6 | 9,678 **±**3,489^**^  5 | 10,232 **±**2,920^***^  8 | 4,750 **±**1,645^***^  11 | 10,283 **±**2,540^***^  12 |
| **18 Hour LPS stimulation**  *TNF-a (pg/ml)*  *n* | 21,735**±** 2,245  33 | 1,082**±** 340^***^  6 | 3,370**±** 1,447^**^  5 | 11,166 **±**3,564  4 | 3,773**±** 1,489^***^  8 | 2,013**±** 1,419^***^  9 | 3,568**±** 1,175^***^  9 |

Data are presented as mean ± standard error. **p<0.005, ***p<0.0005 Vs. HC

HC, Healthy control, ISS, Injury severity score; LPS, Lipopolysaccharide, TNF-α, Tumour necrosis factor-

alpha.
