# Supplementary material for: The impact of trauma relevant concentrations of prostaglandin E2 on the anti-microbial activity of the innate immune system
Source: Front Immunol. 2024 Oct 22;15:1401185. doi: 10.3389/fimmu.2024.1401185 (PMC11535544; doi:10.3389/fimmu.2024.1401185)
Supplement: Supplementary Table 3 — Paired measurements of prostaglandin E2 (PGE2) concentrations and oxidative burst response of E.coli or phorbol 12-myristate 13-acetate (PMA) stimulated neutrophils measured for trauma patients 48-72 hours post-injury. MFI, Mean fluorescence intensity; PGE2, Prostaglandin E2; PMA, phorbol 12-myristate 13-acetate. [file Table3.docx]

**Supplementary Table 3. Paired measurements of prostaglandin E_2_ (PGE_2_) concentrations and oxidative burst response of *E.coli* or phorbol 12-myristate 13-acetate (PMA) stimulated neutrophils measured for trauma patients 48-72 hours post-injury.**

| **Study Subject** | **PGE_2_ (pg/ml)** | **E.coli-induced oxidative burst (MFI)** | **PMA-induced oxidative burst (MFI)** |
| --- | --- | --- | --- |
| **1**  **2**  **3**  **4**  **5**  **6**  **7**  **8** | 1,278  250  386  149  527  991  1,445  715 | 5,704  38,733  9,098  29,738  29,277  11,777  13,736  18,262 | 1,248  30,028  5,886  23,251  12,059  5,041  5,659  13,037 |

MFI, Mean fluorescence intensity; PGE_2_, Prostaglandin E_2_; PMA, phorbol 12-myristate 13-acetate
