# Supplementary material for: The impact of trauma relevant concentrations of prostaglandin E2 on the anti-microbial activity of the innate immune system
Source: Front Immunol. 2024 Oct 22;15:1401185. doi: 10.3389/fimmu.2024.1401185 (PMC11535544; doi:10.3389/fimmu.2024.1401185)
Supplement: Supplementary Table 4 — Comparison of the expression of enzymes and receptors involved in prostaglandin E2 synthesis and signalling in peripheral blood mononuclear cells (PBMCs) and neutrophils isolated from male and female trauma patients. COX-2, Cyclooxygenase-2; cPLA2, cytosolic phospholipase A2; EP2, E prostanoid receptor 2; EP4, E prostanoid receptor 4; mPGES-1, microsomal prostaglandin E synthase-1; PBMCs, Peripheral blood mononuclear cells. Data are presented as expression relative to values recorded for healthy controls. Significant differences in gene expression between males and females are indicated in bold font. [file Table4.docx]

**Supplementary Table 4. Comparison of the expression of enzymes and receptors involved in prostaglandin E2 synthesis and signalling in peripheral blood mononuclear cells (PBMCs) and neutrophils isolated from male and female trauma patients.**

| **PBMCs** | **T=0** | |  | **T=4-12** | |  | **T=48-72** | |  | |
| --- | --- | --- | --- | --- | --- | --- | --- | --- | --- | --- |
|  | **Male** | **Female** | **p** | **Male** | **Female** | **p** | **Male** | **Female** | **p** |  |
|  |  |  |  |  |  |  |  |  |  |  |
| *COX-2* | 7.27± 1.13 | 4.96± 2.90 | 0.33 | 11.12± 1.80 | 18.02± 3.39 | **0.02** | 6.18± 0.69 | 7.29± 1.18 | 0.25 |  |
| *cPLA_2_* | 1.18± 0.31 | 0.85± 0.22 | 0.74 | 2.92± 0.39 | 8.58± 3.20 | 0.07 | 2.57± 0.23 | 3.01± 0.27 | 0.50 |  |
| *mPGES-1* | 5.03± 1.62 | 13.23± 12.22 | 0.73 | 4.29± 1.50 | 4.44± 1.50 | 0.15 | 2.46± 0.64 | 8.55± 5.48 | 0.09 |  |
| *EP2* | 1.01± 0.11 | 0.89± 0.25 | 0.98 | 1.43± 0.17 | 2.20± 0.52 | 0.10 | 1.35± 0.15 | 1.38± 0.37 | 0.90 |  |
| *EP4* | 2.15± 0.28 | 2.90± 0.79 | 0.24 | 1.19± 0.12 | 1.34± 0.48 | 0.92 | 1.33± 0.15 | 1.23± 0.54 | 0.32 |  |
| **Neutrophils** |  |  |  |  |  |  |  |  |  |  |
| *COX-2* | 13.59±  2.92 | 93.48± 84.39 | 0.56 | 4.94± 0.57 | 12.89± 4.72 | 0.23 | 4.83± 0.85 | 4.56± 2.14 | 0.98 |  |
| *cPLA_2_* | 1.38± 0.20 | 0.78± 0.07 | 0.31 | 1.02± 0.12 | 1.15± 0.25 | 0.50 | 0.92± 0.13 | 1.15± 0.42 | 0.49 |  |
| *mPGES-1* | 1.14± 0.17 | 1.48± 0.48 | 0.26 | 1.27± 0.15 | 1.54± 0.38 | 0.26 | 0.33± 0.04 | 0.60± 0.25 | 0.42 |  |
| *EP2* | 1.30± 0.23 | 1.70± 0.18 | 0.06 | 1.26± 0.23 | 19.43± 15.33 | **0.002** | 2.57± 0.58 | 5.03± 2.45 | 0.20 |  |
| *EP4* | 0.93± 0.08 | 0.78± 0.28 | 0.40 | 0.46± 0.06 | 0.68± 0.35 | 0.82 | 0.55± 0.06 | 0.79± 0.32 | 0.59 |  |

COX-2, Cyclooxygenase-2; cPLA_2_, cytosolic phospholipase A2; EP2, E prostanoid receptor 2; EP4, E prostanoid receptor 4; mPGES-1, microsomal prostaglandin E synthase-1; PBMCs, Peripheral blood mononuclear cells. Data are presented as expression relative to values recorded for healthy controls. Significant differences in gene expression between males and females are indicated in bold font.
